# Characterization of Distinct Monocyte Subtypes and Immune Features Associated with HIV, Tuberculosis, and Coronary Artery Disease in a Ugandan Cohort Using Mass Cytometry

**DOI:** 10.20411/pai.v11i1.945

**Published:** 2026-01-29

**Authors:** José Cobeña-Reyes, Celestine N. Wanjalla, Manuel G. Feria, Joshua Simmons, Tecla Temu, Cindy Nochowicz, Sheikh Yasir Arafat, Cissy Kityo, Geofrey Erem, Christopher T. Longenecker, Sandra Andorf, Moises A. Huaman

**Affiliations:** 1 Division of Biomedical Informatics, Cincinnati; Children's Hospital Medical Center, Cincinnati, Ohio; 2 Division of Infectious Diseases, Vanderbilt University Medical Center, Nashville, Tennessee; 3 The Center for AIDS Health Disparities Research, Nashville, Tennessee; 4 Veteran's Health Administration, Tennessee Valley Healthcare System, Nashville, Tennessee; 5 Division of Infectious Diseases, University of Cincinnati College of Medicine, Cincinnati, Ohio; 6 Department of Pathology, Mass General Brigham, Harvard Medical School, Boston, Massachusetts; 7 Department of Biostatistics, Health Informatics, and Data Sciences, College of Medicine, University of Cincinnati, Cincinnati, Ohio; 8 Joint Clinical Research Center, Kampala, Uganda; 9 Makerere University, Kampala, Uganda; 10 Division of Cardiology, Department of Global Health, University of Washington, Seattle, Washington; 11 Division of Allergy and Immunology, Cincinnati Children's Hospital Medical Center, Cincinnati, Ohio; 12 Department of Pediatrics, University of Cincinnati College of Medicine, Cincinnati, Ohio

**Keywords:** Coronary Artery Disease, Tuberculosis, HIV, Monocytes, Mass Cytometry, Immune Dysregulation, CD163, CX3CR1, Biomarker, Unsupervised Clustering, Elastic Net

## Abstract

**Background:**

Coronary artery disease (CAD), tuberculosis (TB), and HIV are major global health concerns. Individuals affected by one or more of these conditions often exhibit chronic inflammation and immune dysregulation, with monocytes playing a central role. Monocyte subsets are known to expand in individuals with HIV, TB, or CAD, but the mechanisms by which these cells contribute to inflammation and immune responses remain poorly understood.

**Methods:**

We employed high-dimensional mass cytometry to characterize monocyte heterogeneity in 61 Ugandan adults with varying combinations of HIV, TB, and subclinical or overt CAD. An integrative approach was used, combining manual gating, unsupervised clustering, and machine learning to identify distinct monocyte phenotypes associated with CAD and TB. Monocyte activation markers soluble CD14 (sCD14) and sCD163 were measured in plasma. CAD was diagnosed by coronary computed tomography angiography. TB was determined by a questionnaire and interferon-gamma release assay (IGRA) testing.

**Results:**

Participants' demographics and clinical characteristics were similar by CAD or HIV/TB status. Median age was 61 years; 37.7% were female. People living with HIV and latent TB or prior active TB had higher sCD14 plasma levels compared with HIV/TB-negative individuals. Individuals with CAD showed reduced surface expression of the scavenger receptor CD163 on non-classical monocytes. Unsupervised clustering further revealed 2 distinct non-classical monocyte subsets associated with disease states: A CD86dim CX3CR1dim CD45RA+ GPR56+ CXCR3+ subset significantly depleted in individuals with CAD, and a CD86+ CX3CR1++ CD45RA++ GPR56− CD38− CXCR3− subset enriched in individuals with latent TB.

**Conclusions:**

These findings underscore the complexity of the monocyte landscape in CAD progression, particularly in regions where HIV and TB are co-endemic. Our study reveals distinct alterations within 2 non-classical monocyte subpopulations associated with CAD and with HIV/TB, offering mechanistic insights that may support the development of precision biomarkers and immune-targeted therapies across these disease contexts.

## INTRODUCTION

HIV, [1] tuberculosis (TB), [2] and cardiovascular diseases [3, 4] are major public health concerns and leading causes of morbidity and mortality worldwide. Individuals residing in Sub-Saharan Africa are particularly vulnerable [5–7], often facing the added challenge of living with one or more of these comorbid conditions simultaneously. People living with HIV (PWH) experience complex immune dysregulation despite appropriate antiretroviral therapy, which may contribute to various comorbidities such as coronary artery disease (CAD) [8] or increased susceptibility to other infectious diseases such as TB [9]. Similarly, people with TB exhibit altered immune responses that can exacerbate immune dysfunction and further elevate CAD risk [10, 11].

Monocytes are cells of the innate immune system that play a critical role in the response to infection and in regulating the immune response. Monocytes are classified based on their CD14 and CD16 expression as classical monocytes (CD14++ CD16-), intermediate monocytes (CD14++ CD16+) and non-classical monocytes (CD14+ CD16++). In PWH, TB, or CAD, monocyte populations undergo significant alterations in both frequency and function [12–15]. In CAD, lower levels of classical monocytes circulating in the blood are usually encountered [16]. Intermediate monocytes tend to aggregate and form monocyte-platelet aggregates in people with ST-elevation myocardial infarction due to coronary atherothrombosis [17]. Further, monocytes infiltrate plaque and differentiate into macrophages (ie, monocyte-derived macrophages). However, these macrophages exhibit a decreased ability to migrate, promoting atherogenesis [18, 19]. In TB infection, CD16+ monocyte subsets are expanded, reflecting alterations in the composition of circulating monocyte populations [20]. The expansion is, however, reversed by anti-TB treatment [20]. Classical monocytes that express inflammatory markers typically increase as the disease progresses, whereas the percentage of non-classical monocytes expressing anti-pathogen infection markers decreases [21]. In PWH, alterations in the distribution of monocyte subsets are also observed, including an increase in intermediate and non-classical monocytes [22]. Interestingly, intermediate monocyte subsets are preferentially infected with HIV due to the facilitation of viral entry [23, 24]. Moreover, there are indications that CD16+ subsets might serve as a reservoir for HIV, complicating viral suppression [25]. Understanding changes in the distribution and phenotype of monocyte subsets is crucial for uncovering disease mechanisms and potential therapeutic targets.

In this work, to characterize monocyte populations in individuals with HIV, TB, and CAD, a cohort of people living with these conditions was studied. We hypothesized that within the spectrum of HIV, TB, and CAD, there would be identifiable monocyte subpopulations that would exhibit distinct phenotypic or functional marker expression patterns. Using mass cytometry data, both manually gated monocyte subtypes and those identified through unsupervised clustering were examined, and their distributions were compared across groups defined by TB, HIV, and CAD status. Additionally, elastic net regression was employed to identify monocyte features most strongly associated with CAD status. This statistical approach enabled the identification of previously undefined immune features that may serve as correlates for disease progression and potential therapeutic intervention.

Given the critical role of monocyte subsets in the regulation of HIV, TB, and CAD, this study aimed to further uncover critical insights into how monocyte populations are altered in HIV, TB and CAD by leveraging high-parameter mass cytometry data and advanced statistical analysis.

## METHODS

### Study Design and Participants

Mass cytometry data from manually gated monocytes isolated from peripheral blood mononuclear cells (PBMCs) of 61 participants were analyzed. All the participants were enrolled at the Joint Clinical Research Center in Kampala, Uganda, in the Ugandan study of HIV effects on the myocardium and atherosclerosis (mUTIMA). For this analysis, the deidentified dataset contained information on individuals living with or without HIV, TB, and CAD. The protocol of the parent study was previously described [26]. The study was approved by the University Hospitals Cleveland Medical Center Institutional Review Board, the Joint Clinical Research Centre Research Ethics Committee, and the Uganda National Council for Science and Technology. All participants signed written informed consent.

TB infection status was defined as latent tuberculosis infection (TBI+) or negative (TB−) based on comprehensive TB symptom questionnaires and interferon-gamma release assay (IGRA) testing (QuantiFERON^®^-TB). Previous active tuberculosis (TBpr) was defined by self-reported history of prior active TB and completion of TB treatment, which was confirmed by review of medical records. HIV status of HIV-negative (HIV−) participants was confirmed with a rapid HIV test (HIV 1/2 STAT-PAK^®^; FDA-approved). The Atherosclerotic Cardiovascular Disease (ASCVD) pooled cohort equations (PCE) were used to calculate 10-year ASCVD risk scores [27]. All participants underwent coronary computed tomography angiography to quantify the presence and severity of CAD. In addition, 2 soluble markers of monocyte activation were measured in plasma from all participants [28]; sCD14 and sCD163, using ELISA (R&D Systems).

Two categories were employed based on the CAD status: A binary classification defined as the presence or absence of coronary plaque (CAD−/+) and a categorization based on burden of disease using the segment involvement score (SIS), an integer-based measure that assigns a value of 1 to each coronary artery segment with detectable atherosclerotic plaque, irrespective of plaque size or degree of luminal obstruction [29]: CAD−, CAD+ minimum (CAD min): ≤ 2 SIS; and CAD+ greater than minimum (CAD+ >min): > 2 SIS. Five study groups were also defined by combining the TB and HIV status as HIV-TB−, HIV-TBI+, HIV+TB−, HIV+TBI+, and HIV+TBpr.

### Mass Cytometry

All the CyTOF experiments were conducted at Vanderbilt University Medical Center through the Center for AIDS Research (CFAR) core. Reagents were purchased from Fluidigm, now Standard BioTools (see Supplementary Table 1 for more details). All samples were run in a Helios CyTOF 3.0 instrument.

A solution of 90% FBS (Fetal Bovine Serum) with 10% DMSO (dimethyl sulfoxide) was used for cryopreservation of PBMCs, as it preserves the expression of markers included in this panel. This panel has been used to stain both cryopreserved and freshly isolated PBMCs, without significant changes in marker expression.

Cryopreserved PBMCs were rapidly thawed in a 37 °C water bath and immediately transferred to 10 mL ice-cold RPMI-1640 (no serum) containing 20 μL Nuclease S7 (Roche) to degrade extracellular nucleic acids. Cells were washed twice by centrifugation at 420 × g for 10 minutes at room temperature (RT) in 10 mL phosphate-buffered saline (PBS; without Ca^2+^/Mg^2+^), decanted, and gently resuspended in the residual supernatant. Viable cells were counted by trypan-blue exclusion on a hemocytometer, and aliquots of 3 × 10^6^ cells were transferred to 5 mL polystyrene tubes (Falcon) for staining.

Cells were washed once in 2 mL PBS/1% bovine serum albumin (BSA; Sigma-Aldrich) at 420 × g × 10 minutes (RT) and then stained with 2 μL Live/Dead Rhodium-103 (Fluidigm; 1:500 final) for 15 minutes at 37 °C. After 2 additional washes in PBS/1% BSA (as above), pellets were tapped dry, then resuspended in 50 μL staining buffer (PBS/1% BSA). A master mix of metal-conjugated surface antibodies (see Supplementary Table 1) and EQ^™^ Four Element Calibration Beads (Fluidigm) was prepared to a volume of 40 μL per sample, brought to ∼100 μL total staining volume with staining buffer, and added to each tube. Samples were incubated for 30 minutes at RT, washed twice in 2 mL PBS (420 × g × 10 minutes, RT), and fixed by adding 20 μL 16% paraformaldehyde (PFA; Thermo Fisher) to 200 μL cells (final 1.6% PFA), incubating 15 minutes at RT.

Fixed cells were washed once in 2 mL PBS (800 × g × 7 minutes, RT), aspirated, and permeabilized by adding 1 mL of ice-cold methanol (MeOH), then stored at –20 °C overnight. Cells were then labeled with 2 μL of 25 μM iridium DNA intercalator (Fluidigm) in the presence of 1.6% PFA (20 μL PFA added to 200 μL cells) for 20 minutes at RT, followed by an overnight incubation at 4 °C.

Immediately before acquisition, cells were washed once in PBS and once in Millipore-grade H_2_O (800 × g × 7 minutes), resuspended at 5 × 10^5^ cells/mL in double-distilled H_2_O, and spiked with equilibration (EQ) beads at a 1:10 bead-to-cell ratio (vortex beads vigorously before use). Finally, cell suspensions were passed through a 35 μm nylon filter (CellTrics, Sysmex) and acquired on the Helios at <500 events.

### Monocyte Phenotyping: Manual Gating

Conventional monocyte subsets were manually gated from PBMCs into total monocyte (TM), classical monocyte (CM), intermediate monocyte (IM), and non-classical monocyte (nCM) categories based on CD14 and CD16 expression, as previously described [6] (Supplementary Figure 1).

For each of the monocyte subsets, several populations were identified by manual gating. For the TM, frequencies of the following populations were determined: CD14+, CD14+ HLA-DR+, CD14+ CX3CR1+, CD14+ CD86+, CD14+ CD163+. For the CM, the following subpopulations were considered: CD14+ CD16-, CD14+ CD16− CX3CR1+, CD14+ CD16− CD86+, CD14+ CD16− CD163+. Subpopulations based on the same markers were studied for IM (CD14+ CD16-, CD14+ CD16− CX3CR1, CD14+ CD16− CD86+, CD14+ CD16− CD163+) and nCM (CD14-CD16+, CD14− CD16+ CX3CR1+, CD14− CD16+ CD86+, CD14− CD16+ CD163+).

Additionally, the Median Signal Intensity (MSI) of markers HLA-DR, CX3CR1, and CD86 was computed for TM, CM, IM, and nCM. MSI of CD163 was computed for TM, IM, and nCM.

### Monocyte Phenotyping: Unsupervised Clustering

FCS files containing the total monocytes (TM) obtained from manual gating were read using the flowCore [30] package from the Bioconductor [31] project, and transformed using the *arcsinh* function with a cofactor of 5 [32]. Ten thousand cells were sampled randomly from each file. If a file contained fewer than 10,000 cells, all cells were used. Of the files, 46 contained more than 10,000 cells, while 15 contained fewer. Unsupervised clustering via FlowSOM [33] with default parameters and a predetermined number of 12 clusters was used to identify phenotypically distinct monocyte populations. All markers were used for unsupervised clustering. For each of the samples, the percentage of cells assigned to each of these clusters (ie, monocyte populations) was calculated.

To determine the marker expression within each monocyte population, scaled median expression values [34] were visualized using a heatmap. To visualize 2D map projections of the cell populations, the stratified CAD/SIS study groups (CAD−, CAD+ min and CAD+ > min), were used. For each group, 80,000 cells were randomly sampled and plotted using Uniform Manifold Approximation Projections (UMAPs) with colors for the populations based on the unsupervised clustering overlayed [35].

### Elastic Net Regularization

The features from manual gating and the population percentages from FlowSOM were used to fit a generalized linear model, incorporating a regularization step through elastic net [36], to identify the features most strongly associated with CAD status. In this model, due to the small sample size, the CAD−/+ classification was used as the predicted variable. In the context of CAD status, negative coefficients from the elastic net model are primarily associated with CAD− (absence of disease), while positive coefficients are associated with CAD+ (presence of disease). Elastic net regularization combines the penalties of Ridge and LASSO regression, enabling both variable selection and shrinkage, which helps reduce overfitting and isolate the most informative features. Alpha values ranging from 0 (Ridge Regression) to 1 (LASSO regression), in increments of 0.1 were tested. For each value of alpha, one hundred values of lambda were tested using a 10-fold cross-validation to select the optimal lambda based on the smallest mean misclassification error (MME). Coefficients of nonzero features, ie, variables retained in the regularized model, were visualized in a bar plot.

### Statistical Analysis

Percentages of populations and MSI of the markers from the manual gating, and the percentage of populations obtained from unsupervised clustering were compared across the study groups using a Kruskal-Wallis test [37]. The *P*-values were adjusted to control the false discovery rate (FDR) using the Benjamini-Hochberg method [38]. For clusters with a significant Kruskal-Wallis test (FDR-adjusted *P* < 0.05), Wilcoxon tests [39] between each pair of study groups were performed. The *P*-value for categorical variables was calculated using Fisher's exact test.

All statistical analyses were performed in R (version 4.4.2). Boxplots show the medians, the 1st and 3rd quartiles. Whiskers represent the minimal and maximal values after excluding outliers, defined as points beyond 1.5 times the interquartile range (IQR) from the 25th or 75th percentile. Individual data points representing each participant are shown. Plotting unless otherwise stated was done using the ggplot2 R package (version 3.5.1). R packages flowCore (version 2.18.0), FlowSOM (version 2.14.0), glmnet (version 4.1-8), ggpubr (version 0.6.0), umap (version 0.2.10.0), corrplot (version 0.95), and ComplexHeatmap (version 2.22.0) were also employed.

## RESULTS

### Study Population and Baseline Characteristics by CAD Satus and HIV/TB Status

A total of 61 participants were initially classified according to coronary artery disease (CAD) status as CAD+ or CAD−. Among CAD+ participants, disease burden was further stratified using the segment involvement score (SIS), which quantifies the number of coronary segments containing atherosclerotic plaque. This resulted in three final groups: CAD− (n=40), CAD+ with minimal involvement (CAD+ min; SIS ≤ 2, n=13), and CAD+ with greater involvement (CAD+ >min; SIS > 2, n=8). Sociodemographic characteristics, HIV history, and clinical variables were largely comparable across CAD and CAD/SIS groups. The only significant difference observed was the 10-year ASCVD risk score, which was highest among CAD+ participants overall (Supplementary Table 2) and particularly elevated in the CAD+ >min subgroup (Table 1).

**Table 1.** Demographics and Clinical Characteristics of the Cohort Using 3 CAD/SIS Groups: CAD−, CAD+ min, and CAD+ >min.

	All patients	CAD−	CAD+ min	CAD+ >min	*P*-value
	N = 61	n = 40 (65.6%)	n = 13 (21.3%)	n = 8 (13.1%)	
**Demographics**					
**Age (years)**	61 (56, 65)	60 (55, 65)	60 (57, 64)	65 (64, 72)	0.068
**Sex: female, n (%)**	23 (37.7)	17 (42.5)	5 (38.5)	1 (12.5)	0.380
Education greater than secondary*	23 (37.7)	21 (46.2)	3 (23.1)	2 (28.6)	0.286
Occupation*					
**Farmer**	18 (30.5)	14 (35.9)	2 (15.4)	2 (28.6)	0.511
**Selling good/Business**	10 (16.9)	7 (17.9)	2 (15.4)	1 (14.3)	
**Teacher/Healthcare/Military/Police**	7 (11.9)	6 (15.4)	1 (7.7)	0 (0.0)	
**Not employed**	11 (18.5)	5 (12.8)	3 (23.1)	3 (42.9)	
**Other**	13 (22.0)	7 (17.9)	5 (38.5)	1 (14.3)	
**Medical history**					
Diabetes*	22 (37.3)	9 (23.1)	8 (61.5)	5 (71.4)	0.557
Hypertension*	51 (86.4)	34 (87.2)	11 (84.6)	6 (85.7)	1
**CVD risk factor**					
**ASCVD risk (10-year)**	9.4 (5.7, 15.2)	10.6 (5.3, 14.3)	8.0 (5.0, 12.7)	11.0 (7.5, 27.1)	0.030
**BMI (pound/in2)**	29.2 (25.6, 33.3)	29.0 (26.2, 32.8)	30.5 (25.5, 34.2)	30.1 (26.6, 35.1)	0.810
**Weight (pounds)**	176.4 (151.0, 196.2)	176.4 (156.5, 196.2)	176.4 (149.9, 194.0)	174.2 (157.6, 191.8)	0.870
**Height (in)**	64.4 (61.8, 66.3)	64.6 (62.0, 67.3)	64.0 (61.0, 64.8)	64.0 (62.7, 65.4)	0.280
Systolic*	146 (129.5, 170.5)	151 (126.5, 170.5)	140.0 (132.0, 165.0)	141.0 (133.5, 162.5)	0.872
Diastolic*	88.0 (78.5, 97.0)	88.0 (79.0, 100.0)	84.0 (80.0, 96.0)	90.0 (77.5, 92.5)	0.898
Total cholesterol*	199.7 (190.2, 230.4)	195.0 (180.2, 231.6)	200.5 (174.1, 213.2)	250.1 (192.3, 279.5)	0.422
LDL*	132.6 (114.1, 157.2)	132.4 (113.3, 160.3)	126.0 (116.0, 135.0)	158.3 (124.1, 195.2)	0.061
HDL*	53.1 (43.1, 64.4)	53.9 (44.2, 66.4)	51.2 (46.2, 60.1)	53.1 (39.3, 55.98)	0.151
Statin*	8 (13.6)	4 (10.3)	2 (15.4)	2 (28.6)	0.3

Continuous variables are presented as median (interquartile range). *P*-values for continuous variables were obtained using the Kruskal-Wallis test. The *P*-value for the only categorical variable was calculated using Fisher's exact test. *P*-values were adjusted for false discovery rate (FDR) using the Benjamini-Hochberg (BH) procedure. None of the participants were current smokers.

* n=59

In parallel, we characterized the study population by HIV and TB status, as these are 2 major infectious diseases that could drive monocyte alterations within our study setting. This yielded 5 HIV/TB categories: HIV-TB− (n=13), HIV-TBI+ (n=22), HIV+TB− (n=9), HIV+TBI+ (n=9), HIV+TBpr (n=8). Overall, no major differences were observed in baseline sociodemographic or clinical characteristics across HIV/TB groups (Table 2). Subgroup analyses within the TB categories (TB−, TBI+, and TBpr) and the HIV categories (HIV+ and HIV− similarly revealed no statistically significant baseline characteristics (Supplementary Tables 3 and 4).

**Table 2.** Demographics and Clinical Characteristics of the Cohort Classified Using 5 HIV/TB Groups: HIV-TB−, HIV-TBI+, HIV+TB−, HIV+TBI+, HIV+TBpr.

	All patients	HIV− TB−	HIV− TBI+	HIV+ TB−	HIV+ TBI+	HIV+ TBpr	*P*-value
	N = 61	n = 13 (21.3)	n = 22 (36.1)	n = 9 (14.8)	n = 9 (14.8)	n = 8 (13.0)	
**Demographics**							
**Age (years)**	61 (56, 65)	60.0 (56.5, 63.8)	62.0 (54.5, 63.8)	65.0 (60.0, 66.0)	56.5 (54.3, 59.0)	62.5 (60.3, 65.0)	0.25
**Sex: female, n (%)**	23 (37.7)	8 (66.7)	7 (31.8)	2 (22.2)	3 (33.4)	3 (37.6)	0.260
Education greater than secondary*	23 (37.7)	6 (50.0)	8 (36.4)	4 (44.4)	4 (50.0)	1 (12.5)	0.475
Occupation*							0.566
**Farmer**	18 (30.5)	3 (25.0)	6 (27.3)	3 (33.3)	3 (37.5)	3 (37.5)	
**Selling good/Business**	10 (16.9)	3 (25.0)	4 (18.2)	1 (11.1)	1 (12.5)	1 (12.5)	
**Teacher/Healthcare/Military/Police**	7 (11.9)	0 (0.0)	4 (18.2)	0 (0.0)	2 (25.0)	1 (12.5)	
**Not employed**	11 (18.5)	0 (0.0)	5 (22.7)	3 (33.3)	1 (12.5)	2 (25.0)	
**Other**	13 (22.0)	6 (50.0)	3 (13.6)	2 (22.2)	1 (12.5)	1 (12.5)	
**Medical history**							
Diabetes*	22 (37.3)	5 (41.7)	7 (31.8)	1 (11.1)	0 (0.0)	3 (37.5)	0.182
Hypertension*	51 (86.4)	10 (83.3)	19 (86.4)	8 (88.9)	7 (87.5)	7 (87.5)	1
**CVD risk factor**							
**ASCVD risk (10-year)**	9.4 (5.7, 15.2)	15.3 (5.4, 24.8)	9.7 (4.9, 12.7)	10.6 (7.8, 12.1)	7.5 (4.3, 9.8)	9.8 (7.5, 33.7)	0.380

**BMI (pound/in2)**	29.2 (25.6, 33.3)	28.2 (26.9, 32.0)	31.8 (27.6, 34.2)	26.1 (25.3, 29.2)	28.1 (25.6, 34.0)	29.6 (27.5, 34.1)	0.551
**Weight (pounds)**	176.4 (151.0, 96.2)	177.5, (154.9, 85.7)	174.2 (159.3, 98.4)	152.1 (129.0, 85.2)	174.2 (157.1, 87.4)	189.6 (167.8, 99.0)	0.590
**Height (in)**	64.4 (61.8, 66.3)	65.4 (62.4, 68.2)	63.8 (61.8, 65.1)	62.2 (60.2, 65.4)	66.1 (63.6, 66.7)	65.2 (62.9, 66.5)	0.480
Systolic*	146 (129.5, 70.5)	149.5 (124.0, 66.5)	142.0 (130.2, 71.8)	141.0 (122.0, 54.0)	143.0 (129.5, 73.0)	156.5 (143.8, 76.8)	0.608
Diastolic*	88.0 (78.5, 97.0)	87.5 (73.8, 94.5)	89.0 (80.5, 101.5)	84.0 (80.0, 90.0)	89.5 (79.0, 96.5)	87.5 (75.3, 98.3)	0.934
Total cholesterol*	199.7 (190.2, 30.4)	193.7 (172.0, 14.7)	193.2 (180.3, 21.1)	250.1 (218.0, 61.2)	203.9 (185.3, 14.9)	187.3 (170.6, 25.0)	0.055
LDL*	132.6 (114.1, 57.2)	124.8 (114.2, 55.5)	133.2 (113.1, 49.9)	160.8 (149.7, 81.1)	129.6 (121.0, 50.0)	131.4 (108.2, 39.5)	0.39
HDL*	53.1 (43.1, 64.4)	46.3 (39.2, 57.3)	55.7 (50.4, 65.9)	60.4 (45.5, 73.7)	52.0 (40.8, 66.07)	47.2 (39.0, 55.9)	0.711
HIV characteristics*							
**HIV duration**	13.9 (11.9, 15.5)	NA	NA	13.9 (12.2, 15.1)	8.3 (8.0, 11.3)	14.0 (13.3, 16.7)	0.392
**ART duration**	11.9 (8.8, 13.8)	NA	NA	12.8 (11.3, 14.1)	7.4 (7.3, 9.7)	12.1 (9.4, 13.1)	0.589
**Nadir CD4+**	187 (135.5, 36.8)	NA	NA	162.0 (67.0, 264.0)	223.0 (199.0, 58.5)	183.5 (135.5, 78.2)	0.397
**Statin use**	8 (13.6)	1 (8.3)	4 (18.2)	1 (11.1)	0 (0.0)	2 (25.0)	0.64

Continuous variables are presented as median (interquartile range). *P*-values for continuous variables were obtained using the Kruskal-Wallis test, adjusted for false discovery rate (FDR) using the Benjamini-Hochberg (BH) procedure. The *P*-value for the only categorical variable was calculated using Fisher's exact test. *P*-values were adjusted for false discovery rate (FDR) using the Benjamini-Hochberg (BH) procedure. None of the participants were current smokers.

* n=59

### Soluble Markers of Monocyte Activation by CAD Status and HIV/TB Status

Soluble monocyte activation markers showed no significant differences across CAD groups (sCD14: *P* = 0.396 and sCD163: *P* = 0.951). Interestingly, significant differences emerged when comparing soluble monocyte activation markers, particularly sCD14, by HIV/TB status. Individuals living with HIV had higher sCD14 levels compared to HIV− participants (*P* = 0.000498, Supplementary Figure 2, top left panel). Within the TB groups, participants in the TBpr group exhibited elevated sCD14 levels relative to both TBI+ and TB− groups (*P* = 0.002; Supplementary Figure 2, top right panel). No statistically significant differences in sCD163 were observed across HIV and TB groups (Supplementary Figure 2, bottom panels). Additional differences were observed across the combined TB/HIV groups (Supplementary Figure 3), overall showing a gradual increase of sCD14 through the HIV/TB spectrum, with the lowest sCD14 plasma levels in the HIV-TB− group, and the highest sCD14 levels in the HIV+TBpr group. Across the CAD groups, sCD163 and sCD14 levels did not differ significantly (Supplementary Figure 4).

### CAD+ >Min Participants Show Lower MSI of CD163 Than CAD− and CAD+ Min in Manually Gated nCM

We then moved to our planned characterization of monocyte populations by CAD status and HIV/TB status. We hypothesized that at least one monocyte subpopulation would differ significantly in abundance across the 5 HIV/TB groups as well as across the CAD groups. It was further hypothesized that such a population would exhibit distinct phenotypic or functional marker expression patterns compared with all other study groups.

Frequencies of different subpopulations and MSI of several markers in TM, CM, IM, and nCM, respectively, based on manual gating, were compared across the CAD−/+ and CAD/SIS groups (Supplementary Figures 5 – 12). Statistically significant differences were observed in the nCM (Supplementary Figure 8, FDR-adjusted *P* = 0.020). Individuals in the CAD+ >min group had a lower MSI of CD163 compared to both the CAD− and CAD+ min groups (*P* = 0.023 in both cases). No statistically significant differences in the manually gated populations were observed across the 5 TB/HIV nor in the CAD−/+ groups (Supplementary Figures 9 – 16).

### Unsupervised Clustering Identifies Distinct Subpopulations

**Figure 1.**
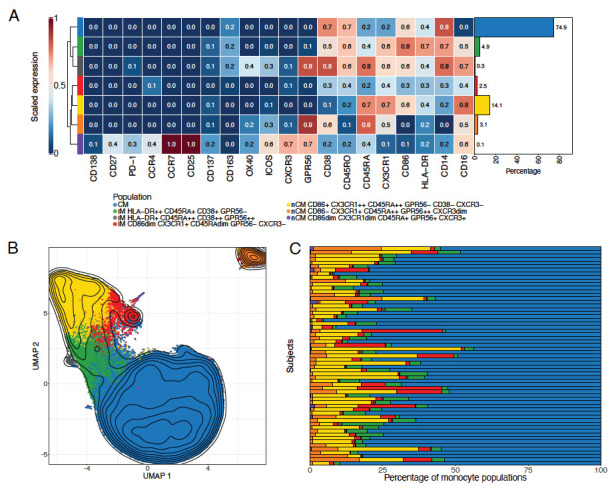
**Monocyte subpopulations identification via FlowSOM and UMAP.** A) Scaled median marker expressions of the monocyte populations found using FlowSOM. Seven populations (1 classical, 3 intermediate, and 3 non-classical monocytes) were identified after merging similar populations (see Supplementary Figure 13). The bar plot on the right indicates the percentage of the population with respect to all cells, ie, all manually gated monocytes used during clustering, in all the samples. B) Uniform Manifold Approximation and Projections (UMAPs) of 240,000 monocytes randomly selected from the cells used in the unsupervised clustering. The populations obtained from the FlowSOM analysis were overlaid. C) Percentage of the monocyte populations obtained from FlowSOM in each of the samples in the cohort. CM, classical monocytes; IM intermediate monocytes; nCM, non-classical monocytes

Unsupervised clustering via FlowSOM was used to separate the total monocytes into 12 clusters. Scaled median marker expressions were used to classify these into 2 CM, 5 IM, and 5 nCM populations (Supplementary Figure 17). The 2 CM populations (clusters 1 and 3) were merged into a single population, as relevant markers such as CD14, CD16, and HLA-DR were similarly expressed. Four IM subpopulations (clusters 4 and 5; and clusters 2 and 9) were also combined into 2 populations. Three nCM populations (clusters 6, 7, and 10) were merged due to similar characteristics. This resulted in a final list of 7 clusters, corresponding to 1 CM, 3 IM, and 3 nCM populations, for downstream analyses (Figure 1). The heatmap of scaled marker expression of each population, along with their percentages in all cells, is shown in Figure 1A. Visualization of the clustering results in a UMAP (Figure 1B) showed that most populations clustered closely together. However, 2 of the nCM populations were slightly (purple population in the plot) or markedly (orange population) separated from the rest.

The CM population comprises the highest percentage of monocytes (74.9%), which is also visually observed in most samples (Figure 1C). Among the 3 IM populations, the HLA-DR++ CD45RA+ CD38+ GPR56− is the largest (4.9%), followed by the CD86dim CX3CR1+ CD45RAdim GPR56− CXCR3− (2.5%) and by HLA-DR+ CD45RA++ CD38++ GPR56++ (0.3%). The nCM portion includes the CD86+ CX3CR1++ CD45RA++ GPR56− CD38− CXCR3+ population (14.1%), which is the largest after the CM. The other 2 nCM populations are CD86− CX3CR1+ CD45RA++ GPR56++ CXCR3dim (3.1%), which is located separately from the other populations in the corner of the UMAP (Figure 1B), possibly due to the lack of expression in CD14 and CD86; and the population CD86dim CX3CR1dim CD45RA+ GPR56+ CXCR3+ (0.1%).

### Percentages of 2 Different nCM Populations from the Unsupervised Clustering Differ Between the Different CAD/SIS and TB/HIV Study Groups

Next, the frequencies of the 7 populations derived from unsupervised clustering, calculated as percentages of the total monocyte population used for clustering, were compared across our study groups.

**Figure 2.**
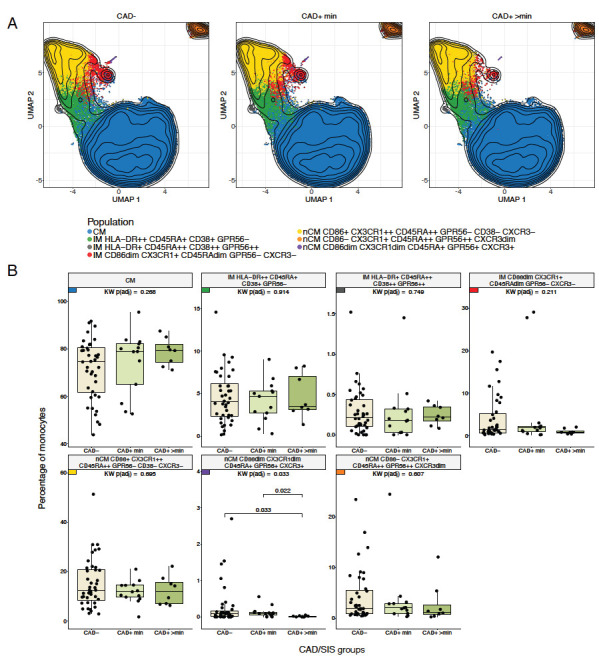
**Differences in monocyte subpopulations obtained with FlowSOM across CAD/SIS Groups.** A) Uniform Manifold Approximation and Projections (UMAPs) stratified by CAD/SIS study groups. 80000 cells were randomly selected per CAD/SIS group, and clusters from the FlowSOM analysis were overlaid. Contour lines are of all cells sampled. B) Statistical differences of percentages of cells for each monocyte population obtained from unsupervised clustering by FlowSOM across the three CAD/SIS study groups. Kruskal-Wallis (KW) tests were performed as an omnibus test to assess overall differences across the groups and *P*-values were adjusted for False Discovery Rate (FDR) using the Benjamini-Hochberg method. The population nCM CD86dim CX3CR1dim CD45RA+ GPR56+ CXCR3+ was statistically significant (FDR-adjusted *P* = 0.033) and was further analyzed by post-hoc Wilcoxon tests across the 3 groups. The percentage in the CAD+ >min study group was statistically lower than the CAD+ min (*P* = 0.022) and CAD− (*P* = 0.033).

Hypothesis testing was conducted to identify differences across the CAD/SIS groups (CAD−, CAD+ min, and CAD+ >min groups). The CD86dim CX3CR1dim CD45RA+ GPR56+ CXCR3+ nCM cluster (purple population in the UMAPs, Figure 2A) showed a statistically significant difference in the percentage of cells among the 3 study groups (FDR-adjusted *P* = 0.033). In post hoc analyses, participants from the CAD+ >min group had lower proportions of this population compared to the CAD− (*P* = 0.033) and CAD+ min groups (*P* = 0.022, Figure 2B). CAD− and CAD+ min participants showed comparable proportions of this population among total monocytes. There were no significant differences across the CAD−/+ groups (Supplementary Figure 18).

**Figure 3.**
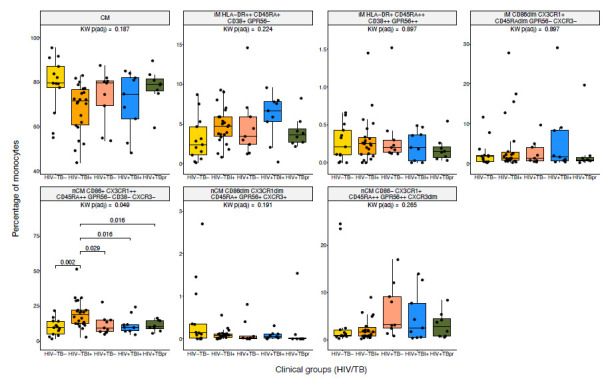
**Differences in monocyte subpopulations obtained with FlowSOM across HIV/TB Groups.** Statistical differences of percentages of cells for each monocyte population obtained from unsupervised clustering by FlowSOM across the 5 HIV/TB study groups. Kruskal-Wallis (KW) tests were performed as an omnibus test and *P*-values were adjusted for False Discovery Rate (FDR) using Benjamini-Hochberg method. Among the populations tested, only nCM CD86+ CX3CR1++ CD45RA++ GPR56− CD38-CXCR3− reached statistical significance (FDR-adjusted *P* < 0.05) and was further subjected to post-hoc Wilcoxon tests across the 5 groups. The small colored rectangles on top left corners follow the colors used in the UMAPs in Figures 1 and 2.

Then, the proportion of each of the 7 FlowSOM-derived populations was compared across the 5 HIV/TB study groups. In this analysis, a different nCM population showed statistically significant differences between the groups. The nCM cluster characterized as CD86+ CX3CR1++ CD45RA++ GPR56− CXCR3− (yellow population in the UMAPs, Figures 1 and Supplementary Figure 16) was present at significantly higher proportions in the HIV-TBI+ group compared to all the other 4 TB/HIV clinical groups (Figure 3). Interestingly, the same trend was observed in the manual gating results (Supplementary Figure 16), where the HIV-TBI+ group showed the highest median of both percentage of CX3CR1+ and MSI of CX3CR1 in nCM. The trends might be a sign of inflammation in people who are TBI+, in agreement with previous research [20].

### Non-Classical Monocyte CX3CR1 Expression and Subset Composition Correlate With Cardiovascular Risk

To identify monocyte characteristics associated with CAD, elastic net regression analysis was performed using CAD−/+ status as the predicted variable. The regression model included 32 features (MSI and percent of populations) obtained by manually gating, and the percentages of the 7 populations identified in the FlowSOM clustering.

**Figure 4.**
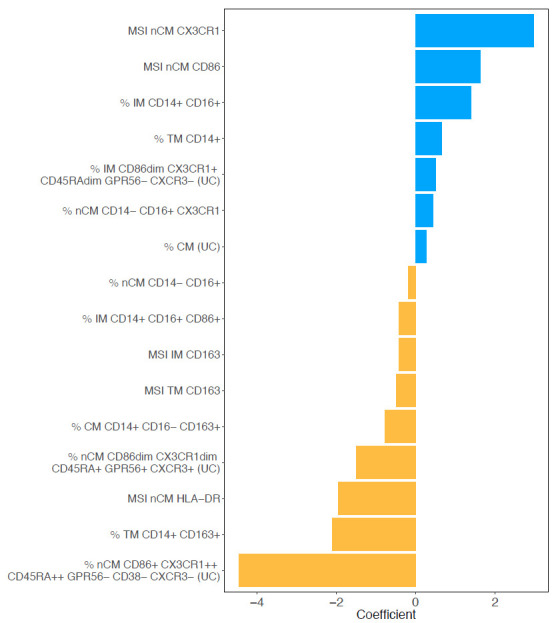
**Monocyte features associated with CAD status via regularized regression.** Nonzero features obtained from elastic net regression and their coefficients associated with the CAD+/− status. The coefficients were obtained after testing alpha values from 0 to 1 in 0.1 increments. The alpha value that led to the smallest MSE was 0.6 (elastic net regularization). Manual gating and unsupervised clustering variables were used as features. (UC) indicates that the variable was obtained from unsupervised clustering. A total of 16 variables were retained as important predictors of the CAD status after regularization. Seven and nine variables were positively and negatively associated, respectively.

During model tuning, an alpha value of 0.6 was selected based on evaluation across 100 lambda values per alpha, as this combination yielded the lowest mean misclassification error (Supplementary Figure 20). As a result, the fitted model was equivalent to an elastic net penalization that combines L1 and L2 regularization, shrinking coefficients of less important features to zero and effectively selecting a sparse set of predictors. Figure 4 shows the coefficients of the nonzero features retained in the final model.

The top 3 variables that were associated with CAD+ were the MSI of CX3CR1 and CD86 in nCM, and the percent of IM CD14+ CD16+. The top 3 that were associated with CAD− were the percent of nCM CD86+ CX3CR1++ CD45RA++ GPR56− CD38− CXCR3− identified in the unsupervised clustering, the percent of TM CD14+ CD163+, and the MSI of HLA-DR in nCM. The TM CD14+ CD163 agrees with the results obtained from the unsupervised clustering shown in Figure 2.

## DISCUSSION

In this study, mass cytometry and advanced statistical analysis were employed to perform immunophenotyping of monocyte populations and study their associations with infectious and inflammatory diseases, specifically HIV, tuberculosis, and cardiovascular disease. Elastic net regression to identify key features that are most strongly correlated with CAD−/+ status was also applied.

The higher sCD14 levels in individuals with HIV likely reflect chronic monocyte activation driven by microbial translocation and systemic inflammation. The stepwise increase across TB categories suggests that *M. tuberculosis* exposure further amplifies this activation. The combined gradient (HIV+TBpr > HIV+TBI+ > HIV+ TB− > HIV-TBI+ > HIV−TB−) points to additive effects of HIV and TB, supporting sCD14 as an indicator of cumulative innate immune activation in co-endemic settings [40].

The analysis of the manually gated data showed a statistically significant difference across the CAD/SIS groups regarding the median signal intensity of CD163 in nCM (Figure S8). CD163 has previously been shown to be a potential predictor of mortality and heart failure in people with CAD [41]. Previous studies have indicated that CD163 is characteristic of anti-inflammatory monocytes and has been identified as a marker related to CAD [42]. The downregulation of CD163 MSI in nCMs among individuals with CAD may occur under specific conditions. For instance, inflammation could promote CD163 shedding, producing sCD163 while reducing detectable CD163 surface expression [43]. Furthermore, in vitro studies show CD163 downregulation in monocytes and macrophages has been associated with inflammation mediators such as TNF-α, IFN-γ, and LPS [44].

When comparing the populations obtained from the unsupervised clustering across the CAD/SIS groups, the nCM population CD86dim CX3CR1dim CD45RA+ GPR56+ CXCR3+ was basically absent in the CAD+ >min group. CD86 is a co-stimulatory molecule and plays a crucial role in T cell activation by binding to CD28 on T cells, providing the necessary second signal for full T cell activation. CD86 expression suggests that the subset might be involved in interaction with T cells, likely promoting an immune response [45]. CX3CR1 is a chemokine receptor predominantly expressed on nCMs, which actively patrol the vascular endothelium under both homeostatic and inflammatory conditions [46]. CD45RA is a characteristic marker of naïve T cells and is less well-characterized in monocyte populations [47]. GPR56 is an adhesion protein-coupled receptor that primarily functions as an inhibitor in natural killer cells. Studies involving GPR56+ monocytes are limited. However, due to the similarities between GPR56 and EMR2, it is likely that the function of GPR56+ is similar to EMR2+ monocytes that are involved in the activation and migration of innate cells during systemic inflammation responses [48]. CXCR3 is another chemokine receptor present in monocytes that can infiltrate CAD-related lesions. CXCR3+ monocytes are associated with plaque instability and adverse cardiovascular events [49]. It is possible that depletion of this nCM population with low expression of CD86 and CX3CR1 could be an indication of increased relative abundance of highly expressing CD86 and CX3CR1 nCM in the circulation of people with CAD. Alternatively, the lower percentage of nCM with low expression of CD86 and CX3CR1 in people with CAD might indicate that most of these cells may have already migrated to the tissues and are no longer in the bloodstream, thus contributing to atherosclerotic lesion development.

Across the TB/HIV groups, the HIV-TBI+ group exhibited a greater percentage of nCM co-expressing CD86+ and CX3CR1++ compared to the other groups. The CX3CR1 upregulation suggests enhanced chemotactic potential and tissue patrolling capacity, possibly reflecting a more activated, vigilant monocyte phenotype even in latent infection [6]. Further, a recent study in mice infected with TB showed that the absence of chemokine receptors CX3CR1 was associated with an altered positioning of derived dendritic cells in mediastinal lymph nodes [50]. The expression of CD86 suggests a role of this population in antigen presentation. Previously it has been reported that circulating nCMs from HIV-TBI+ individuals exhibit a higher production of interleukin (IL)-6 and tumor necrosis factor (TNF)-α pro-inflammatory cytokines, compared to HIV-TBI− control individuals [6]. Future studies can define the functional capacity of this newly classified CD86+ CX3CR1++ CXCR3+ nCM subpopulation and their role in TB infection control and persistent inflammation.

It is noteworthy that in the HIV–TBI+ group, we found an enrichment of nCM subsets highly expressing CX3CR1 and CD86, whereas the CAD+>min group had a significant decrease of nCMs with low CD86 and CX3CR1 expression. These 2 nCM groups appear to reflect distinct biological trajectories. In the HIV-TBI+ group, the nCM subset shows elevated CX3CR1 and CD86 expression, consistent with a patrolling/monitoring phenotype. In contrast, the nCM CD86dim CX3CR1dim CD45RA+ GPR56+ CXCR3+ cluster is significantly reduced in individuals in the CAD+ >min group. This depletion suggests that CAD may be associated with lower circulating frequencies of CD86dim CX3CR1dim nCMs, potentially reflecting accelerated recruitment, activation, or apoptosis of this patrolling subset in the setting of chronic endothelial injury and systemic inflammation [51–53]. Given that CAD is characterized by active recruitment of CX3CR1+ CD86+ nCMs to inflamed vasculature, the CD86dim CX3CR1dim subset may also be mobilized out of circulation. Moreover, the CAD inflammatory milieu is known to shift monocytes toward more activated phenotypes (eg, CD86+) while selectively depleting less-activated populations [52]. This finding aligns with prior observations that chronic inflammatory diseases, including CAD, remodel hematopoiesis and skew circulating myeloid cell distributions. Collectively, these findings suggest that CX3CR1− and CD86-expressing nCM subsets participate in shared inflammatory pathways that may actively contribute to disease pathogenesis in both chronic infectious and cardiovascular settings. In CAD, depletion of circulating CD86^dim CX3CR1^dim nCMs may reflect enhanced recruitment of these cells to inflamed endothelium, where CX3CR1-mediated adhesion, antigen presentation, and local cytokine production could promote vascular inflammation and atherosclerotic lesion development. In contrast, enrichment of CX3CR1^+ CD86^+ non-classical monocytes in HIV-TBI+ individuals suggests a sustained patrolling and antigen-presenting phenotype that may contribute to persistent immune activation and immunopathology during latent infection. Further validation in longitudinal cohorts will be required to determine whether these subsets can inform disease monitoring, prognosis, or targeted immunomodulatory strategies.

The nonzero features obtained in the elastic net regression provided information about the relevant features associated with the CAD−/+ status. For instance, among the variables strongly associated with CAD+ status, the MSI of CX3XR1 in nCM had the coefficient with the greatest absolute value, which is an indication of a strong immune response and a sign of inflammation in HIV/TB coinfections [54]. The next positive coefficient was the MSI of CD86 in nCM. It has been shown that expression of CD86 increases as CAD progresses [55]. The CD86+ nCM are potentially geared toward antigen presentation and T-cell stimulation [56]. The percentage of IMs was also positively associated with CAD+. IM are pro-inflammatory and atherogenic [56]. Also, IMs are associated with increased carotid intima-media thickness, a marker of atherosclerosis, and predicted cardiovascular risk even in general population cohorts [57]. Among the features that were identified as important predictors of CAD−, the nCM population CD86+ CX3CR1++ CD45RA++ GPR56− CD38− CXCR3− was the most strongly inversely associated with CAD. Moderate CD86 expression in nCMs suggests a role in immune surveillance, rather than driving full activation and inflammation [58]. In general, CX3CR1 is strongly expressed on nCMs. CX3CR1+ nCM typically patrol the endothelium and contribute to tissue repair and vascular stability [46, 59]. The other 2 variables associated with CAD− are the percentage of TM CD14+ CD163+ and the MSI of HLA-DR in nCM. The first of these findings suggests that people in the CAD− group have higher percentages of CD163+monocytes. These cells are likely anti-inflammatory [60]. Angiotensin or context-specific studies link high expression of HLA-DR to monocytes with enhanced antigen presentation and immune surveillance traits, especially in nCMs. One study in HIV populations found that CCR9dim HLA-DR+ nCM were associated with absence of subclinical atherosclerosis, suggesting their HLA-DR+ phenotype is protective [61].

There are several limitations in this study. For instance, the total number of participants in the sample was small for some of the study groups. Therefore, there might not have been enough power to detect subtle differences in monocyte biomarkers between all groups. Moreover, our cohort includes individuals who live with multiple comorbid conditions, making the analysis even more challenging, a common limitation of human-based clinical and translational studies. The limited sample size does not allow for a detailed analysis or adjustment of all combinations of conditions simultaneously. Still, our identification of specific monocyte subpopulations associated with TB and CAD can be further investigated and validated in future studies. The unsupervised clustering analysis was performed on the manually gated monocyte population, which limited the number of cells that could be included per sample. We randomly selected 10,000 cells per sample or used all cells if the sample contained less than 10,000 cells. This was the case for 15 samples, and the minimum number of cells in a sample was 1,333. To address potential variability based on the limited sample size, we performed repeated runs of the unsupervised clustering.

The distinct monocyte subpopulations found in this study deserve more study to understand how these populations interact with the diseases as they evolve. This study provides a framework to investigate the functionality of unusual monocyte populations in diseases that affect a large percentage of the human population.

## SUPPLEMENTARY DATA

Supplementary materials are available at the *Pathogens and Immunity* website. Supplementary data may be provided by the authors to benefit the reader. Supplementary data are not copyedited and are the sole responsibility of the authors. Questions or comments related to supplementary materials should be addressed to the corresponding author.
