# Supplementary material for: Characterization of Distinct Monocyte Subtypes and Immune Features Associated with HIV, Tuberculosis, and Coronary Artery Disease in a Ugandan Cohort Using Mass Cytometry
Source: Pathog Immun. 2026 Jan 29;11(1):14–38. doi: 10.20411/pai.v11i1.945 (PMC12867109; doi:10.20411/pai.v11i1.945)
Supplement: Supplementary Tables and Figures [file pai-11-014-s01.pdf]

PATHOGENS AND IMMUNITY
IN ASSOCIATION WITH CASE WESTERN RESERVE UNIVERSITY AND THE RICHARD J. FASENMYER FOUNDATION

Supplementary Table 1. CyTOF Antibody Panel for Immune Cell Phenotyping

Marker	Clone	Metal Tag	Catalog Number	Vendor
CD45	HI30	89Y	3089003B	Fluidigm/Standard BioTools
CD16	3G8	209Bi	3209002B	Fluidigm/Standard BioTools
CD14	RMO52	148Nd	3148010B	Fluidigm/Standard BioTools
HLA-DR	L243	174Yb	3174001B	Fluidigm/Standard BioTools
CD86	IT2.2	150Nd	3150020B	Fluidigm/Standard BioTools
CX3CR1	*K0124E1*	172Yb	92J046172	Fluidigm/Standard BioTools
CD45RA	HI100	153Eu	3153001B	Fluidigm/Standard BioTools
CD45RO	UCHL1	149Sm	3149001B	Fluidigm/Standard BioTools
CD38	HIT2	144Nd	3144014B	Fluidigm/Standard BioTools
GPR56	4C3	154Sm	N/A	Conjugated at VUMC
CXCR3	G025H7	156Gd	3156004B	Fluidigm/Standard BioTools
ICOS	C398.4A	151Eu	3151020B	Fluidigm/Standard BioTools
OX40	ACT35	158Gd	3158012B	Fluidigm/Standard BioTools
CD163	EDHu-1	147Sm	3147021D	Fluidigm/Standard BioTools
CD137	4B4-1	173Yb	3173015B	Fluidigm/Standard BioTools
CD25	2A3	169Tm	3169003B	Fluidigm/Standard BioTools
CCR7	G043H7	159Tb	3159003A	Fluidigm/Standard BioTools
CCR4	L291H4	175Lu	3175035A	Fluidigm/Standard BioTools
PD-1	EH12.2H7	155Gd	3155009B	Fluidigm/Standard BioTools
CD27	O323	167Er	3167002B	Fluidigm/Standard BioTools
CD138	DL-101	168Er	3168009B	Fluidigm/Standard BioTools
Viability Marker	—	103Rh	201103A	Fluidigm/Standard BioTools
DNA Intercalator	---	Ir191/193	201192B	Fluidigm/Standard BioTools

Cobeña-Reyes J, Wanjalla CN, Feria MG, Simmons J , Temu T , Nochowicz C , Arafat SA, Kityo C , Erem G , Longenecker CT, Andorf S, Huaman MA. Characterization of Distinct Monocyte Subtypes and Immune Features Associated with HIV, Tuberculosis, and Coronary Artery Disease in a Ugandan Cohort Using Mass Cytometry. *Pathogens and Immunity*. 2025;11(1):14-38. doi: 10.20411/pai.v11i1.945

PATHOGENS AND IMMUNITY
IN ASSOCIATION WITH CASE WESTERN RESERVE UNIVERSITY AND THE RICHARD J. FASENMYER FOUNDATION

Supplementary Figure 1. Manual gating strategy sample

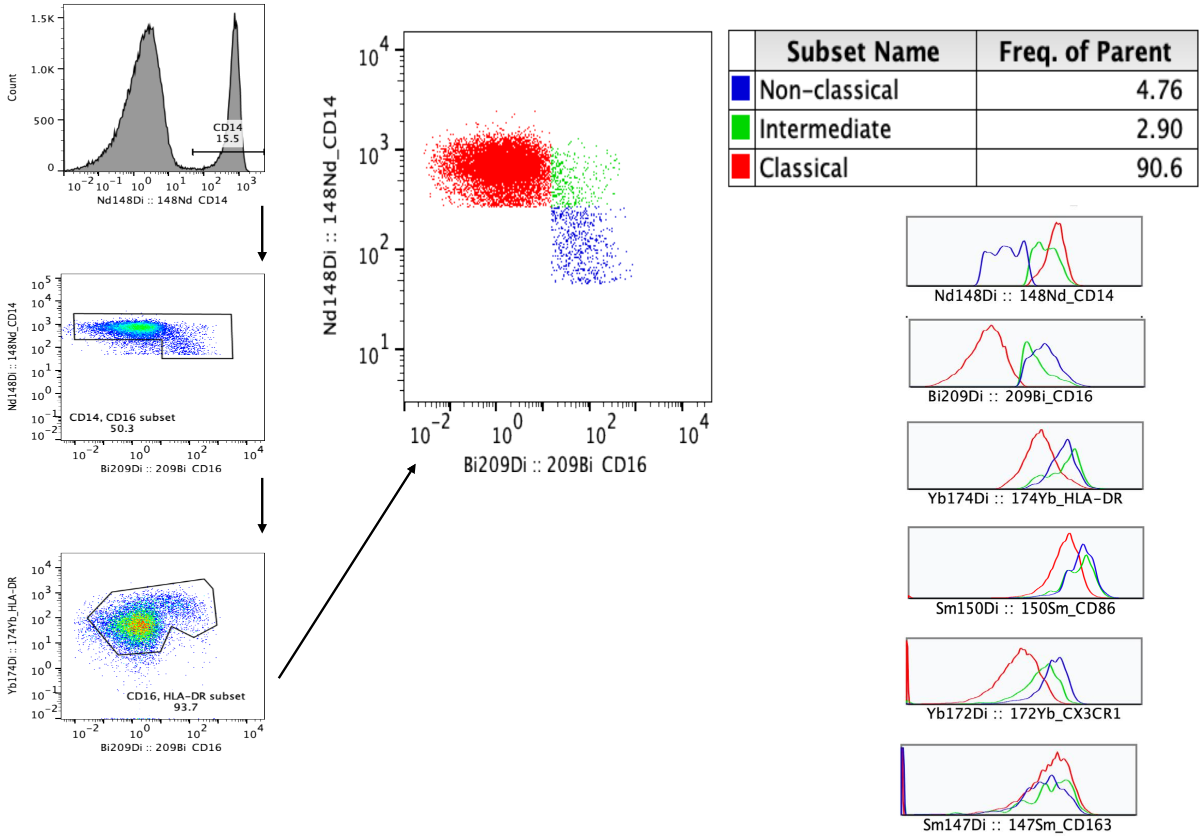

Cobeña-Reyes J, Wanjalla CN, Feria MG, Simmons J , Temu T , Nochowicz C , Arafat SA, Kityo C , Erem G , Longenecker CT, Andorf S, Huaman MA. Characterization of Distinct Monocyte Subtypes and Immune Features Associated with HIV, Tuberculosis, and Coronary Artery Disease in a Ugandan Cohort Using Mass Cytometry. *Pathogens and Immunity*. 2025;11(1):14-38. doi: 10.20411/pai.v11i1.945

PATHOGENS AND IMMUNITY
IN ASSOCIATION WITH CASE WESTERN RESERVE UNIVERSITY AND THE RICHARD J. FASENMYER FOUNDATION

Supplementary Table 2. Demographics and Clinical Characteristics of the Cohort Using the CAD-/+ Classification

	All patients N = 61	CAD- n = 40 (65.6%)	CAD+ n = 21 (34.4%)	*P*-value
Demographics				
Age [years]	61 (56, 65)	60 (55, 65)	63 (58.5, 66)	0.16
Sex: female, n (%)	23 (37.7)	17 (42.5)	6 (28.6)	0.320
Education greater than secondary*	23 (37.7)			
Occupation*				0.29
Farmer	18 (30.5)	14 (35.9)	4 (20.0)	
Selling good/Business	10 (16.9)	7 (17.9)	3 (15.0)	
Teacher/Healthcare/Military/Police	7 (11.9)	6 (15.4)	1 (5.0)	
Not employed	11 (18.5)	5 (12.8)	6 (30.0)	
Other	13 (22.0)	7 (17.9)	6 (30.0)	
Medical history*				
Diabetes	22 (37.3)	9 (23.10	7 (35.0)	1
Hypertension	51 (86.4)	34 (87.2)	17 (85)	0.329
CVD risk factor				
ASCVD risk (10-year)	9.4 (5.7, 15.2)	10.6 (5.3, 14.3)	8.4 (6.5, 18.1)	**0.009**
BMI [pound/in^2]	29.2 (25.6, 33.3)	29.0 (26.2, 32.8)	30.3, (25.4, 34.6)	0.660
Weight [pounds]	176.4 (151.0, 196.2)	176.4 (156.5, 196.2)	170.5 (149.9, 195.1)	0.640
Height [in]	64.4 (61.8, 66.3)	64.6 (62.0, 67.3)	64.0, (61.6, 65.0)	0.140
Systolic*	146 (129.5, 170.5)	151.0 (126.5, 170.5)	140.5 (131.8, 166.5)	0.636
Diastolic*	88.0 (78.5, 97.0)	88.0 (79.0, 100.5)	87.0 (78.5, 95.25	0.683
Total cholesterol*	199.7 (190.2, 230.4)	195.0 (180.2, 231.6)	200.5 (205.4, 226.6)	0.3785
LDL*	132.6 (114.1, 157.2)	132.4 (113.3, 160.3)	133.2 (120.2, 151.1)	0.12
HDL*	53.1 (43.1, 64.4)	53.9 (44.2, 66.4)	52.5 (40.9, 57.8)	0.911
Statin*	8 (13.6)	4 (10.3)	4 (20.0)	0.424

Continuous variables are presented as median (interquartile range). P-values for continuous variables were obtained using the Kruskal-Wallis test, adjusted for false discovery rate (FDR) using the Benjamini-Hochberg (BH) procedure. The *P*-value for the only categorical variable was calculated using Fisher's exact test. P-values were adjusted for false discovery rate (FDR) using the Benjamini-Hochberg (BH) procedure. None of the participants were current smokers. * n = 59

Cobeña-Reyes J, Wanjalla CN, Feria MG, Simmons J , Temu T , Nochowicz C , Arafat SA, Kityo C , Erem G , Longenecker CT, Andorf S, Huaman MA. Characterization of Distinct Monocyte Subtypes and Immune Features Associated with HIV, Tuberculosis, and Coronary Artery Disease in a Ugandan Cohort Using Mass Cytometry. *Pathogens and Immunity*. 2025;11(1):14-38. doi: 10.20411/pai.v11i1.945

PATHOGENS AND IMMUNITY
IN ASSOCIATION WITH CASE WESTERN RESERVE UNIVERSITY AND THE RICHARD J. FASENMYER FOUNDATION

Supplementary Table 3. Demographics and Clinical Characteristics of the Cohort Classified Using the 3 TB Groups in the Cohort

	**All patients**<br>**N = 61**	**TB-**<br>**n = 22 (36.1)**	**TBI+**<br>**n = 31 (50.8)**	**TBpr**<br>**n = 8 (13.1)**	**p-value**
Demographics					
Age [years]	61 (56, 65)	60 (57.0, 66.0)	59.5 (54.3, 63.0)	62.5 (60.3, 65.0)	0.332
Sex: female, n (%)	23 (37.7)	10 (47.6)	10 (33.3)	3 (37.5)	0.573
Education greater than secondary*	45 (76.3)	10 (47.6)	12 (40.0)	1 (12.5)	0.248
Occupation*					0.324
Farmer	18 (30.5)	6 (28.6)	9 (30.0)	3 (37.5)	
Selling good/Business	10 (16.9)	4 (19.0)	5 (16.7)	1 (12.5)	
Teacher/Healthcare/Military/Police	7 (11.9)	0 (0.0)	6 (20.)	1 (12.5)	
Not employed	11 (18.5)	3 (14.3)	6 (20.0)	2 (25.0)	
Other	13 (22.0)	8 (38.1)	4 (13.3)	1 (12.5)	
Medical history*					
Diabetes	22 (37.3)	6 (28.6)	7 (23.3)	3 (37.5)	0.667
Hypertension	51 (86.4)	18 (85.7)	26 (86.7)	7 (87.5)	1
CVD risk factor					
ASCVD risk (10-year)	9.4 (5.7, 15.2)				
BMI [pound/in^2]	29.2 (25.6, 33.3)	31.2 (27.2, 34.2)	27.7 (25.3, 31.8)	29.6 (27.5, 34.1)	0.332
Weight [pounds]	176.4 (151.0, 196.2)	160.9 (147.7, 185.2)	174.2 (159.3, 198.4)	189.6 (167.8, 199.0)	0.380
Height [in]	64.4 (61.8, 66.3)	64.6 (61.8, 68.1)	64.0 (61.9, 66.1)	65.2 (63.0, 66.5)	0.744
Systolic *	146 (129.5, 170.5)	146.0 (124.0, 164.0)	142.0 (130.0, 173.2)	156.5 (143.8, 176.8)	0.311
Diastolic*	88.0 (78.5, 97.0)	84.0 (79.0, 92.0)	89.0 (80.5, 99.5)	87.5 (75.3, 98.3)	0.803
Total cholesterol*	199.7 (190.2, 230.4)	213.2 (192.4, 250.1)	195.6 (180.3, 221.0)	187.3 (170.6, 225.0)	0.429
LDL*	132.6 (114.1, 157.2)	149.7 (121.6, 166.8)	131.1 (113.8, 150.1)	132.6 (114.1, 157.2)	0.834
HDL*	53.1 (43.1, 64.4)	48.3 (43.5, 62.5)	54.6 (49.8, 66.1)	48.4 (43.5, 62.5)	0.246
Statin use*	8 (13.6)	2 (9.5)	4 (13.3)	2 (25.0)	0.513

Continuous variables are presented as median (interquartile range). P-values for continuous variables were obtained using the Kruskal-Wallis test, adjusted for false discovery rate (FDR) using the Benjamini-Hochberg (BH) procedure. The *P*-value for the only categorical variable was calculated using Fisher's exact test. *P*-values were adjusted for false discovery rate (FDR) using the Benjamini-Hochberg (BH) procedure. None of the participants were current smokers. * n = 59

PATHOGENS AND IMMUNITY
In Association with Case Western Reserve University and The Richard J. Fasenmyer Foundation

Supplementary Table 4. Demographics and Clinical Characteristics of the Cohort Classified Using the 3 HIV groups in the Cohort

	All patients<br>N = 61	HIV-<br>N = 33 (54.1)	HIV+<br>N = 26 (45.9)	*P*-value
Demographics				
Age [years]	61 (56, 65)	61 (56.0, 65.0)	60.5 (55.3, 63.8)	0.656
Sex: female, n (%)	23 (37.7)	15 (44.1)	8 (32.0)	0.346
Education greater than secondary*	23 (37.7)	14 (41.0)	9 (36.0)	0.687
Occupation*				0.682
Farmer	18 (30.5)	9 (26.5)	9 (36.0)	
Selling good/Businnes	10 (16.9)	7 (20.6)	3 (12.0)	
Teacher/Healthcare/Military/Police	7 (11.9)	4 (11.8)	3 (12.0)	
Not employed	11 (18.5)	5 (14.7)	6 (24.0)	
Other	13 (22.0)	9 (26.5)	9 (36.0)	
Medical history*				
Diabetes	22 (37.3)	12 (35.3)	4 (16.0)	0.141
Hypertension	51 (86.4)	29 (85.3)	22 (88.0)	1
CVD risk factor				
ASCVD risk (10-year)	9.4 (5.7, 15.2)			
BMI [pound/in^2]	29.2 (25.6, 33.3)	28.6 (25.3, 33.6)	30.6 (27.4, 33.8)	0.462
Weight [pounds]	176.4 (151.0, 196.2)	176.4 (142.2, 198.4)	175.3 (157.1, 193.5)	0.667
Height [in]	64.4 (61.8, 66.3)	64.4 (61.8, 66.3)	64.1 (61.8, 66.1)	0.560
Systolic*	146 (129.5, 170.5)	146.0 (130.0, 170.0)	145.0 (129.2, 169.5)	0.890
Diastolic*	88.0 (78.5, 97.0)	84.0 (79.0, 96.0)	88.5 (78.5, 101.5)	0.539
Total cholesterol*	199.7 (190.2, 230.4)	211.8 (191.4, 234.6)	193.5, 178.4, 217.7)	0.25
LDL*	132.6 (114.1, 157.2)	135.2 (123.2, 160.8)	129.3 (113.1, 154.1)	0.399
HDL*	53.1 (43.1, 64.4)	52.2 (42.6, 63.1)	54.1 (45.2, 64.8)	0.416
Statin use*	8 (13.6)	5 (14.7)	3 (12.0)	1
HIV characteristics*				
HIV duration	13.9 (11.9, 15.5)	13.9 (11.9, 15.5)	NA	NA
ART duration	11.9 (8.8, 13.8)	11.9 (8.8, 13.8)	NA	NA
Nadir CD4+	187 (135.5, 336.8)	187 (135.5, 336.8)	NA	NA

Continuous variables are presented as median (interquartile range). P-values for continuous variables were obtained using the Kruskal-Wallis test, adjusted for false discovery rate (FDR) using the Benjamini-Hochberg (BH) procedure. The *P*-value for the only categorical variable was calculated using Fisher's exact test. P-values were adjusted for false discovery rate (FDR) using the Benjamini-Hochberg (BH) procedure. None of the participants were current smokers. * n = 59

Cobeña-Reyes J, Wanjalla CN, Feria MG, Simmons J , Temu T , Nochowicz C , Arafat SA, Kityo C , Erem G , Longenecker CT, Andorf S, Huaman MA. Characterization of Distinct Monocyte Subtypes and Immune Features Associated with HIV, Tuberculosis, and Coronary Artery Disease in a Ugandan Cohort Using Mass Cytometry. *Pathogens and Immunity*. 2025;11(1):14-38. doi: 10.20411/pai.v11i1.945

PATHOGENS AND IMMUNITY
IN ASSOCIATION WITH CASE WESTERN RESERVE UNIVERSITY AND THE RICHARD J. FASENMYER FOUNDATION

Supplementary Figure 2. Differences in amount of sCD14 and sCD163 across HIV (left) and TB (right) groups (n = 45).

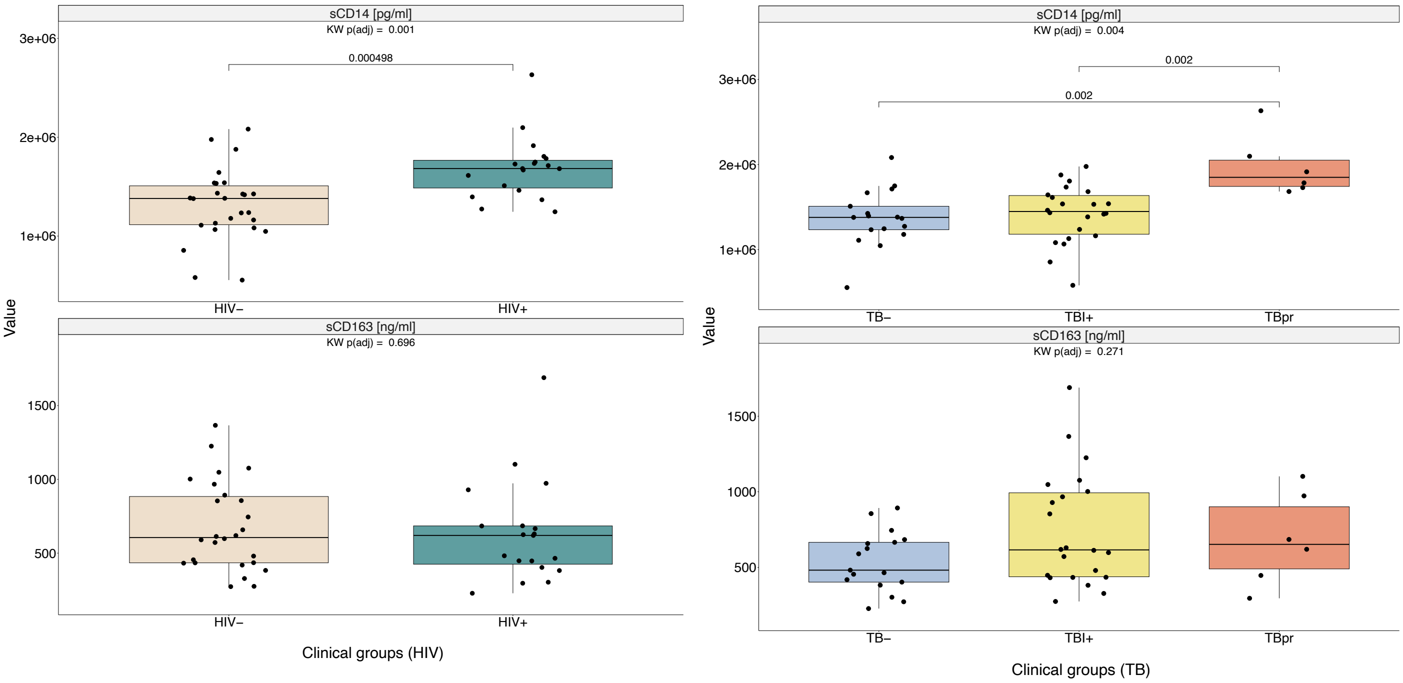

Statistical differences among HIV and TB study groups across sCD14 and sCD163. Kruskal-Wallis (KW) tests were performed, and *P*-values were False Discovery Rate (FDR) adjusted. sCD14 was higher in HIV+ groups ($P = 0.000498$), and in TBpr group compared against the other two TB groups ($P = 0.02$ in both cases).

Cobeña-Reyes J, Wanjalla CN, Feria MG, Simmons J , Temu T , Nochowicz C , Arafat SA, Kityo C , Erem G , Longenecker CT, Andorf S, Huaman MA. Characterization of Distinct Monocyte Subtypes and Immune Features Associated with HIV, Tuberculosis, and Coronary Artery Disease in a Ugandan Cohort Using Mass Cytometry. *Pathogens and Immunity*. 2025;11(1):14-38. doi: 10.20411/pai.v11i1.945

PATHOGENS AND IMMUNITY
IN ASSOCIATION WITH CASE WESTERN RESERVE UNIVERSITY AND THE RICHARD J. FASENMYER FOUNDATION

Supplementary Figure 3. Differences in the amount of sCD14 and sCD163 across HIV/TB groups (n = 45).

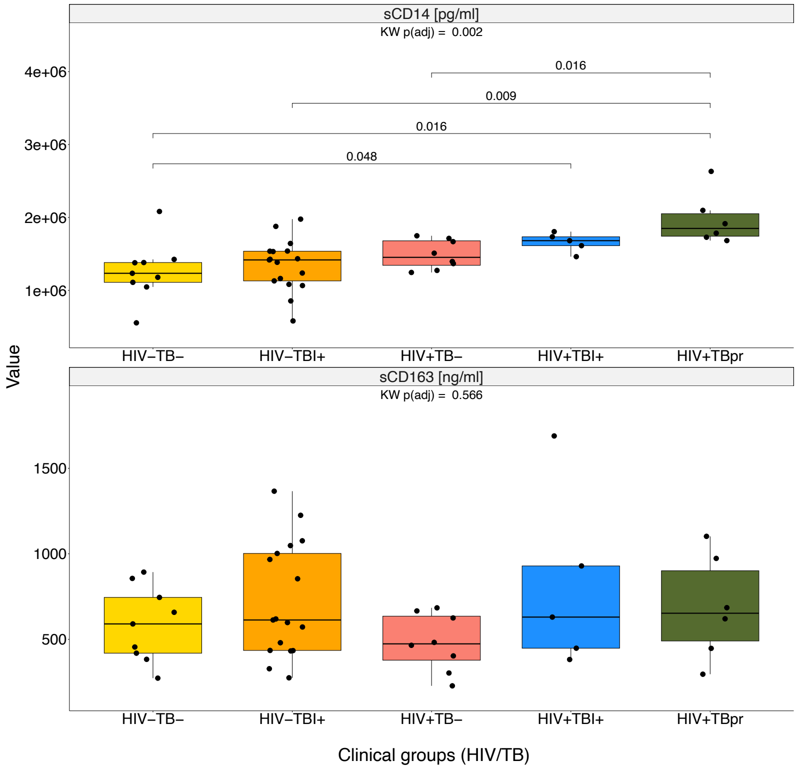

Statistical differences among HIV/TB groups across sCD14 and sCD163. Kruskal-Wallis (KW) tests were performed, and p-values were False Discovery Rate (FDR) adjusted. Overall, sCD14 was statistically significantly different across groups. Medians increased with as HIV-TB < HIV-TBI+ < HIV+TB- < HIV+TBI+ < HIV+TBpr, indicating that HIV+ and TBI+ along with TBpr increased the amount of sCD14.

Cobeña-Reyes J, Wanjalla CN, Feria MG, Simmons J , Temu T , Nochowicz C , Arafat SA, Kityo C , Erem G , Longenecker CT, Andorf S, Huaman MA. Characterization of Distinct Monocyte Subtypes and Immune Features Associated with HIV, Tuberculosis, and Coronary Artery Disease in a Ugandan Cohort Using Mass Cytometry. *Pathogens and Immunity*. 2025;11(1):14-38. doi: 10.20411/pai.v11i1.945

PATHOGENS AND IMMUNITY
IN ASSOCIATION WITH CASE WESTERN RESERVE UNIVERSITY AND THE RICHARD J. FASENMYER FOUNDATION

Supplementary Figure 4. Differences in the amount of sCD14 and sCD163 across CAD groups (n = 16).

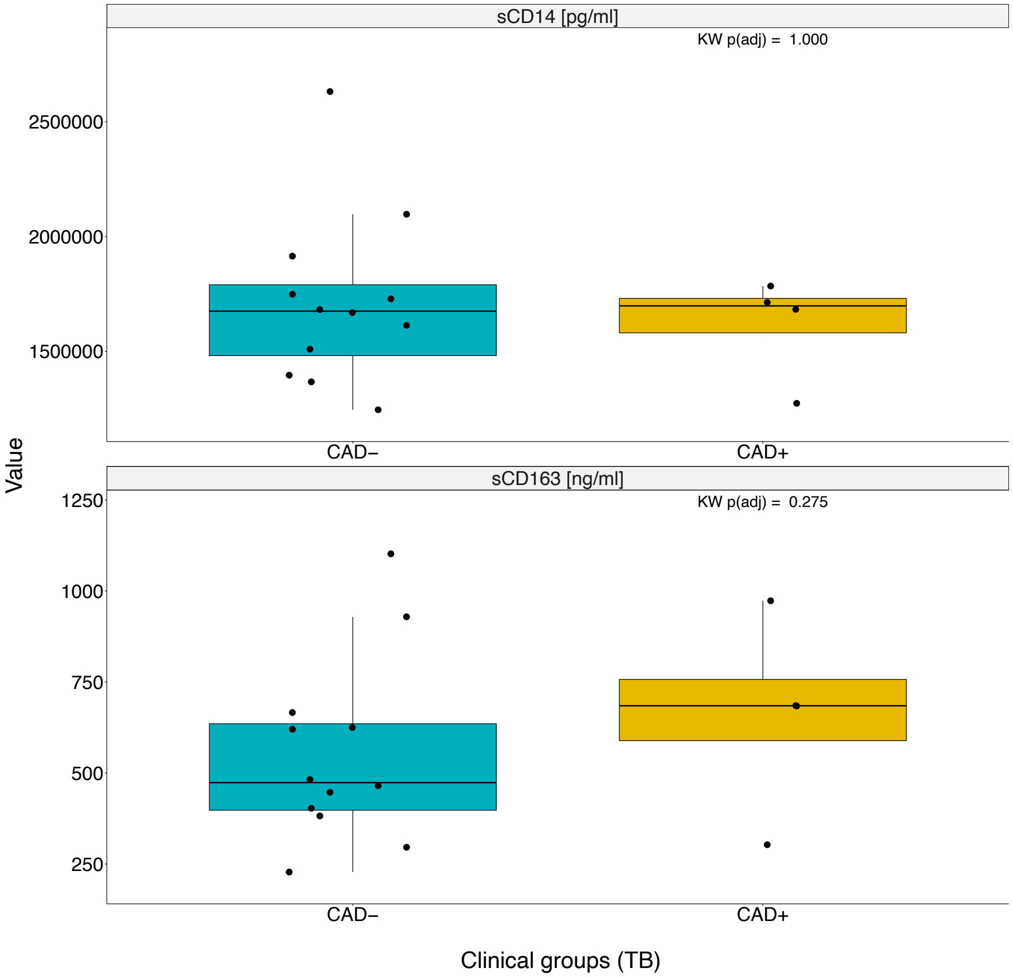

No statistical differences between CAD groups across sCD14 and sCD163 were found. Kruskal-Wallis (KW) tests were performed, and p-values were False Discovery Rate (FDR) adjusted.

Cobeña-Reyes J, Wanjalla CN, Feria MG, Simmons J , Temu T , Nochowicz C , Arafat SA, Kityo C , Erem G , Longenecker CT, Andorf S, Huaman MA. Characterization of Distinct Monocyte Subtypes and Immune Features Associated with HIV, Tuberculosis, and Coronary Artery Disease in a Ugandan Cohort Using Mass Cytometry. *Pathogens and Immunity*. 2025;11(1):14-38. doi: 10.20411/pai.v11i1.945

PATHOGENS AND IMMUNITY
In Association with Case Western Reserve University and The Richard J. Fasenmyer Foundation

Supplementary Figure 5. Differences in total monocytes manually gated populations and MFI across CAD/SIS.

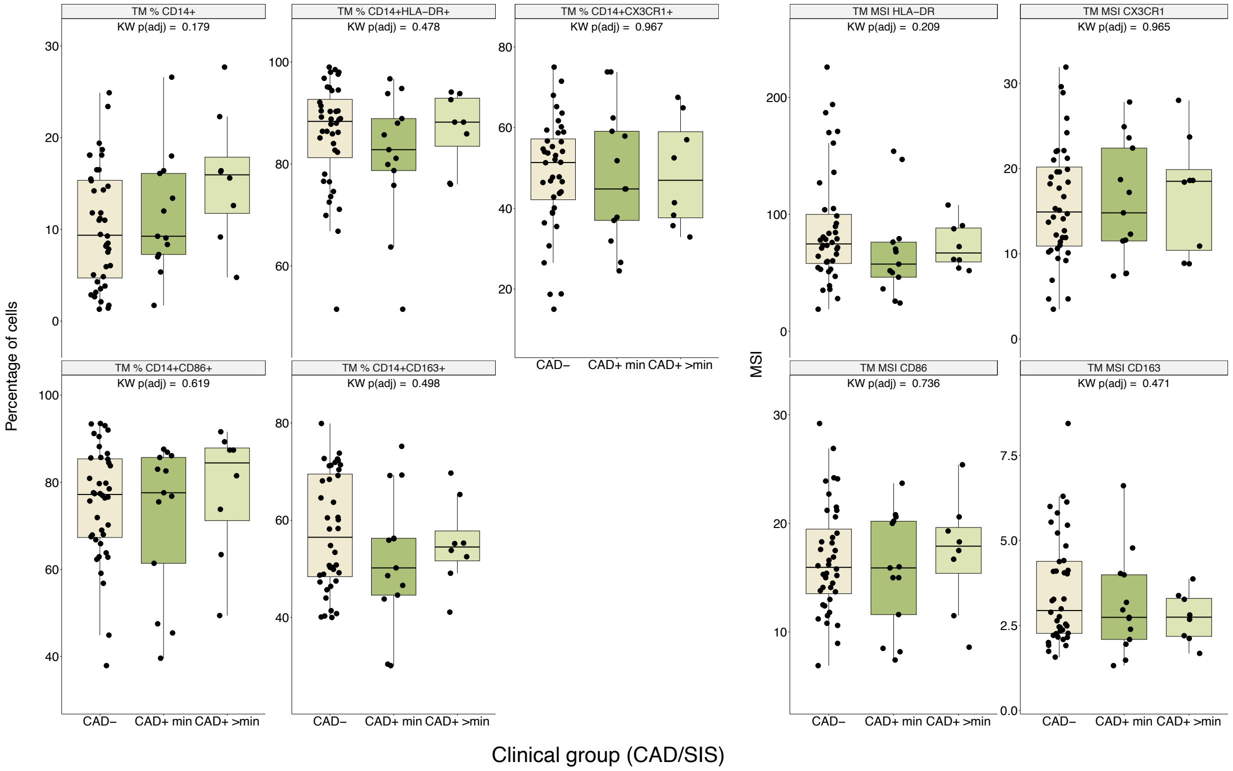

Statistical differences among CAD/SIS study groups across total monocyte (TM) populations and MSI of selected markers. Kruskal-Wallis (KW) tests were performed, and *P*-values were False Discovery Rate (FDR) adjusted. No statistically significant differences were found.

Cobeña-Reyes J, Wanjalla CN, Feria MG, Simmons J , Temu T , Nochowicz C , Arafat SA, Kityo C , Erem G , Longenecker CT, Andorf S, Huaman MA. Characterization of Distinct Monocyte Subtypes and Immune Features Associated with HIV, Tuberculosis, and Coronary Artery Disease in a Ugandan Cohort Using Mass Cytometry. *Pathogens and Immunity*. 2025;11(1):14-38. doi: 10.20411/pai.v11i1.945

PATHOGENS AND IMMUNITY
In Association with Case Western Reserve University and The Richard J. Fasenmyer Foundation

Supplementary Figure 6. Differences in classical monocytes manually gated populations and MFI across CAD/SIS.

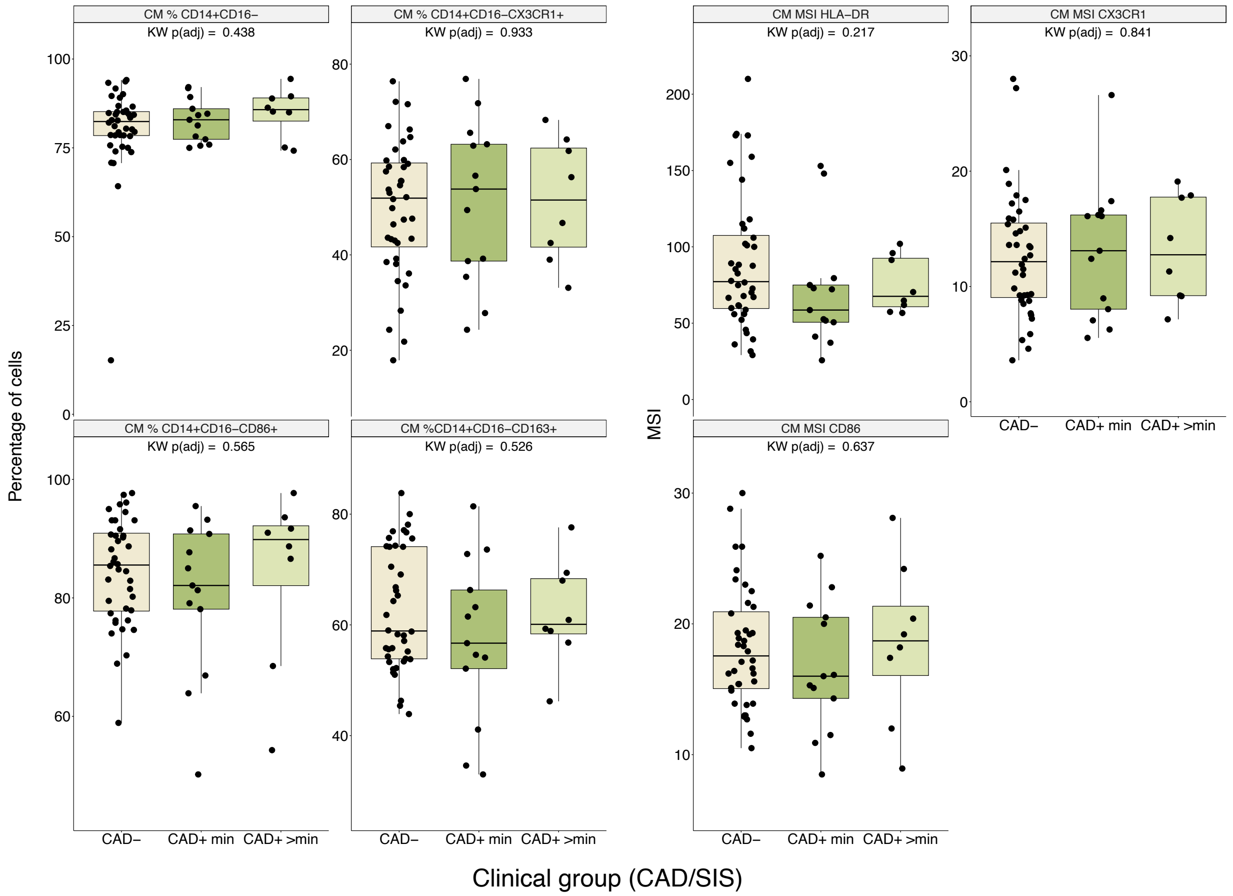

Statistical differences among CAD/SIS study groups across classical monocyte (CM) populations and MSI of selected markers. Kruskal-Wallis (KW) tests were performed, and *P*-values were False Discovery Rate (FDR) adjusted. No statistically significant differences were found.

Cobeña-Reyes J, Wanjalla CN, Feria MG, Simmons J , Temu T , Nochowicz C , Arafat SA, Kityo C , Erem G , Longenecker CT, Andorf S, Huaman MA. Characterization of Distinct Monocyte Subtypes and Immune Features Associated with HIV, Tuberculosis, and Coronary Artery Disease in a Ugandan Cohort Using Mass Cytometry. *Pathogens and Immunity*. 2025;11(1):14-38. doi: 10.20411/pai.v11i1.945

Supplementary Figure 7. Differences in intermediate monocytes manually gated populations and MFI across CAD/SIS.

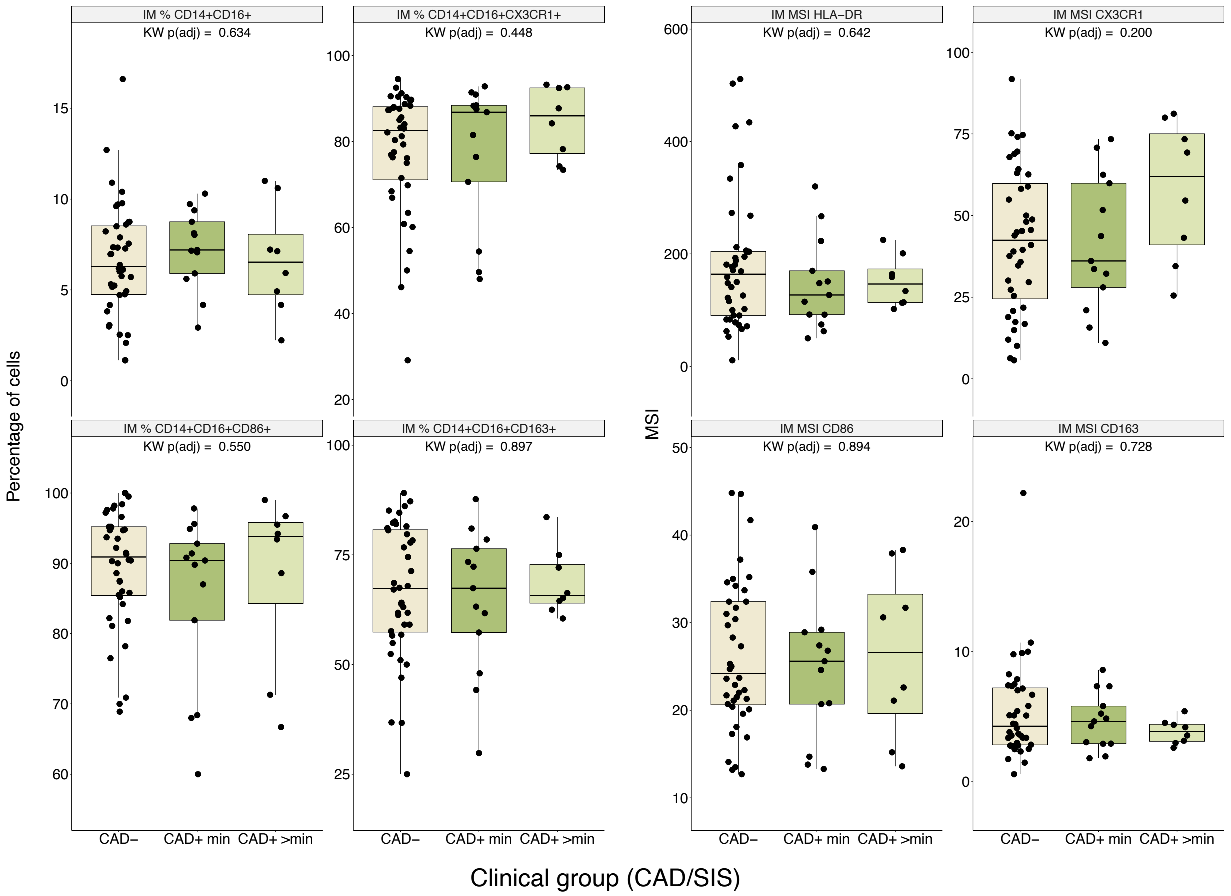

Statistical differences among CAD/SIS study groups across intermediate monocyte (IM) populations and MSI of selected markers. Kruskal-Wallis (KW) tests were performed, and *P*-values were False Discovery Rate (FDR) adjusted. No statistically significant differences were found.

Cobeña-Reyes J, Wanjalla CN, Feria MG, Simmons J , Temu T , Nochowicz C , Arafat SA, Kityo C , Erem G , Longenecker CT, Andorf S, Huaman MA. Characterization of Distinct Monocyte Subtypes and Immune Features Associated with HIV, Tuberculosis, and Coronary Artery Disease in a Ugandan Cohort Using Mass Cytometry. *Pathogens and Immunity*. 2025;11(1):14-38. doi: 10.20411/pai.v11i1.945

PATHOGENS AND IMMUNITY
In Association with Case Western Reserve University and The Richard J. Fasenmyer Foundation

Supplementary Figure 8. Differences in non-classical monocytes manually gated populations and MFI across CAD/SIS.

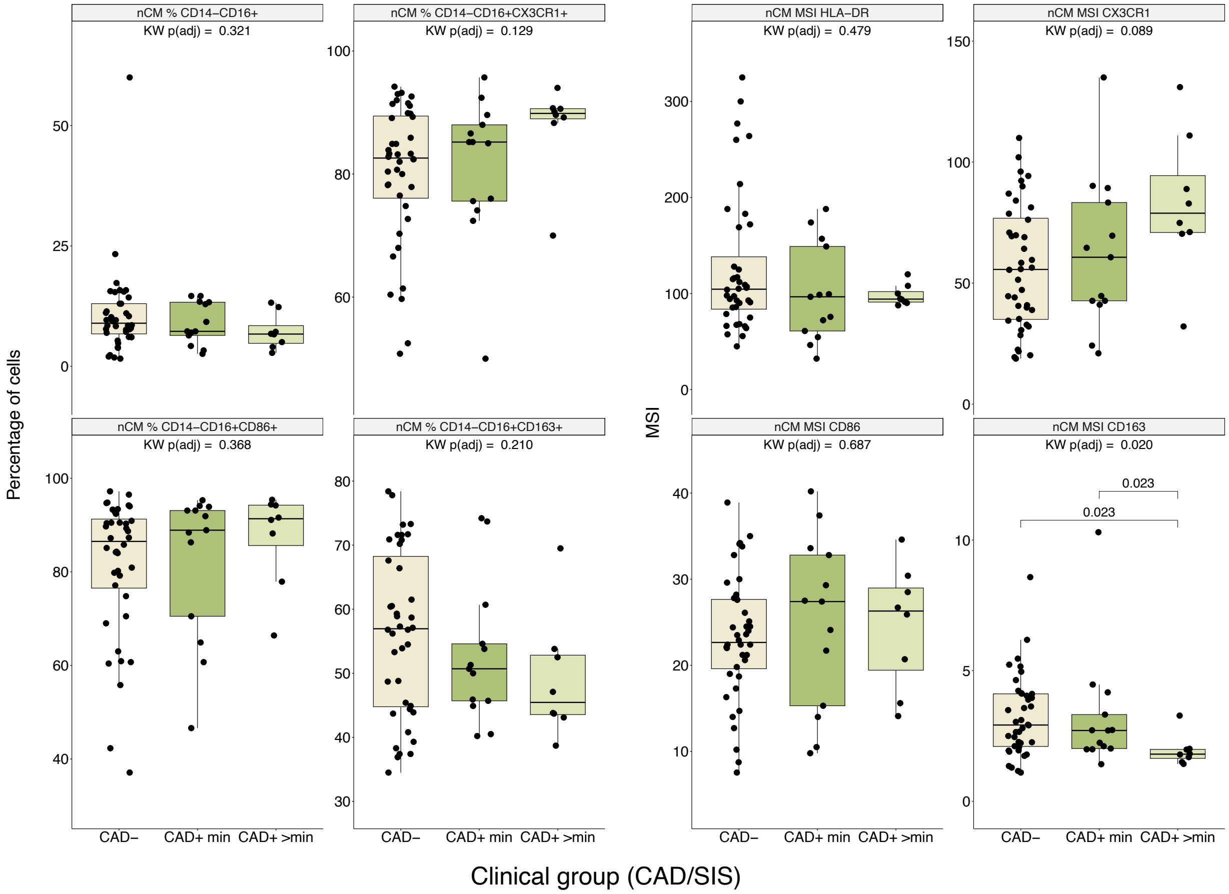

Statistical differences among CAD/SIS study groups across non-classical monocyte (nCM) populations and MSI of selected markers. Kruskal-Wallis (KW) tests were performed and the *P*-values adjusted for False Discovery Rate (FDR) using Benjamini-Hochberg method. Only statistically significant (FDR-adjusted $P < 0.05$) difference was found in the MSI of CD163 in nCM. In post-hoc analyses using Wilcoxon tests, CAD+ >min had significantly lower MSI compared to the other two groups.

Cobeña-Reyes J, Wanjalla CN, Feria MG, Simmons J , Temu T , Nochowicz C , Arafat SA, Kityo C , Erem G , Longenecker CT, Andorf S, Huaman MA. Characterization of Distinct Monocyte Subtypes and Immune Features Associated with HIV, Tuberculosis, and Coronary Artery Disease in a Ugandan Cohort Using Mass Cytometry. *Pathogens and Immunity*. 2025;11(1):14-38. doi: 10.20411/pai.v11i1.945

PATHOGENS AND IMMUNITY
IN ASSOCIATION WITH CASE WESTERN RESERVE UNIVERSITY AND THE RICHARD J. FASENMYER FOUNDATION

Supplementary Figure 9. Differences in total monocytes manually gated populations and MFI across CAD-/+.

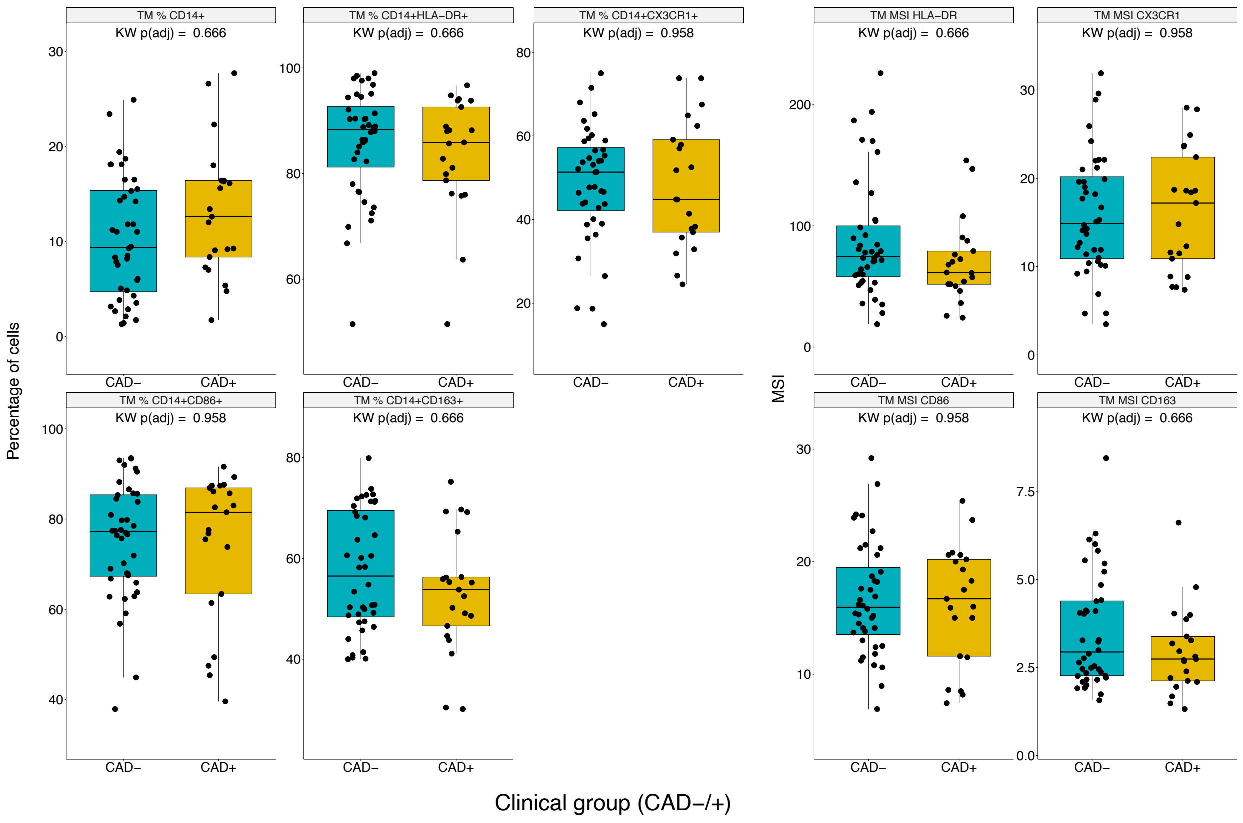

Statistical differences between CAD-/+ across total monocytes (TM) populations and MFI of selected markers. Kruskal-Wallis (KW) tests were performed, and *P*-values were for False Discovery Rate adjusted. No statistically significant differences were found.

Cobeña-Reyes J, Wanjalla CN, Feria MG, Simmons J , Temu T , Nochowicz C , Arafat SA, Kityo C , Erem G , Longenecker CT, Andorf S, Huaman MA. Characterization of Distinct Monocyte Subtypes and Immune Features Associated with HIV, Tuberculosis, and Coronary Artery Disease in a Ugandan Cohort Using Mass Cytometry. *Pathogens and Immunity*. 2025;11(1):14-38. doi: 10.20411/pai.v11i1.945

PATHOGENS AND IMMUNITY
IN ASSOCIATION WITH CASE WESTERN RESERVE UNIVERSITY AND THE RICHARD J. FASENMYER FOUNDATION

Supplementary Figure 10. Differences in classical monocytes manually gated populations and MFI across CAD-/+.

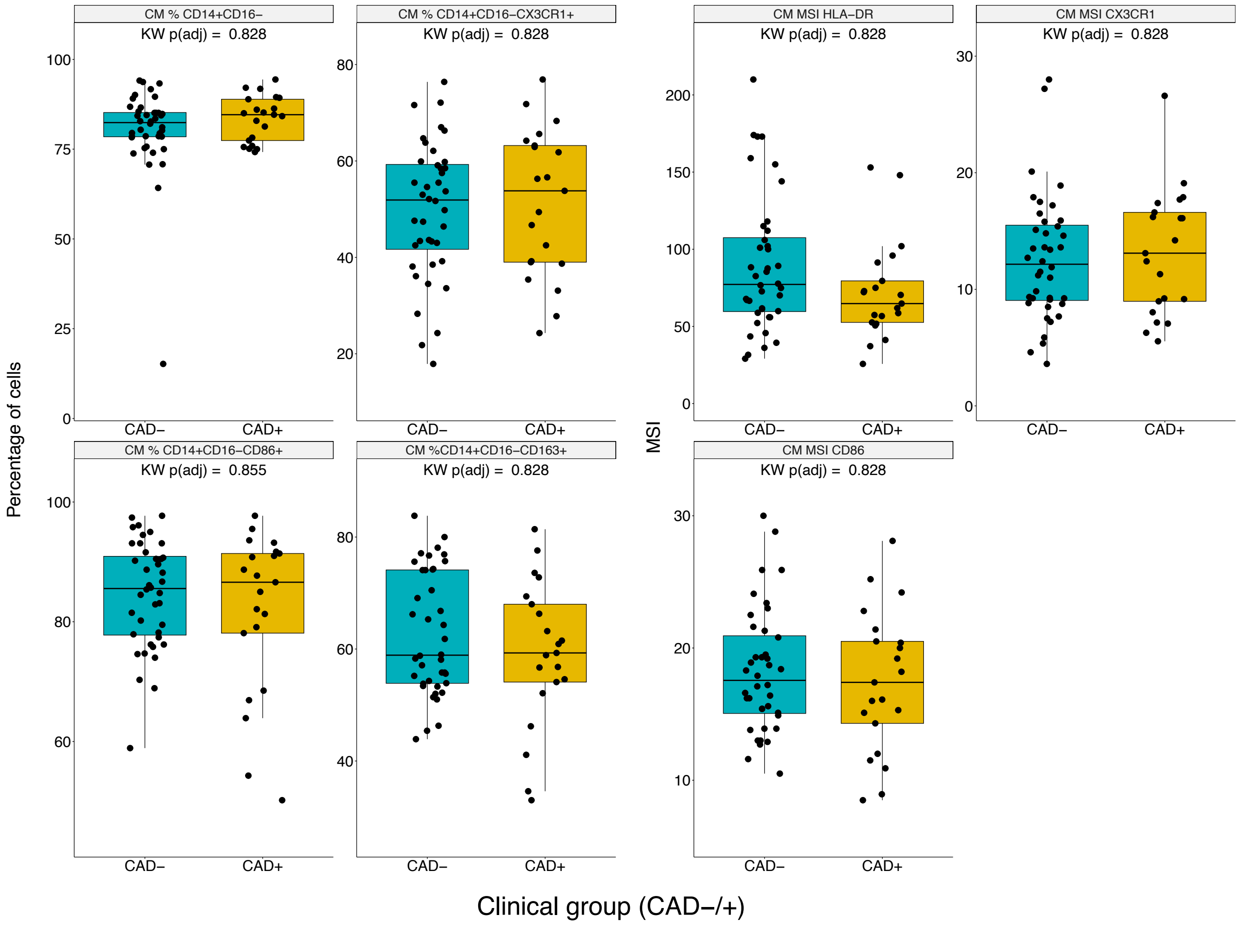

Statistical differences between CAD-/+ across total classical monocyte (CM) populations and MFI of selected markers. Kruskal-Wallis (KW) tests were performed, and *P*-values were for False Discovery Rate adjusted. No statistically significant differences were found.

Cobeña-Reyes J, Wanjalla CN, Feria MG, Simmons J , Temu T , Nochowicz C , Arafat SA, Kityo C , Erem G , Longenecker CT, Andorf S, Huaman MA. Characterization of Distinct Monocyte Subtypes and Immune Features Associated with HIV, Tuberculosis, and Coronary Artery Disease in a Ugandan Cohort Using Mass Cytometry. *Pathogens and Immunity*. 2025;11(1):14-38. doi: 10.20411/pai.v11i1.945

PATHOGENS AND IMMUNITY
IN ASSOCIATION WITH CASE WESTERN RESERVE UNIVERSITY AND THE RICHARD J. FASENMYER FOUNDATION

Supplementary Figure 11. Differences in intermediate monocytes manually gated populations and MFI across CAD-/+.

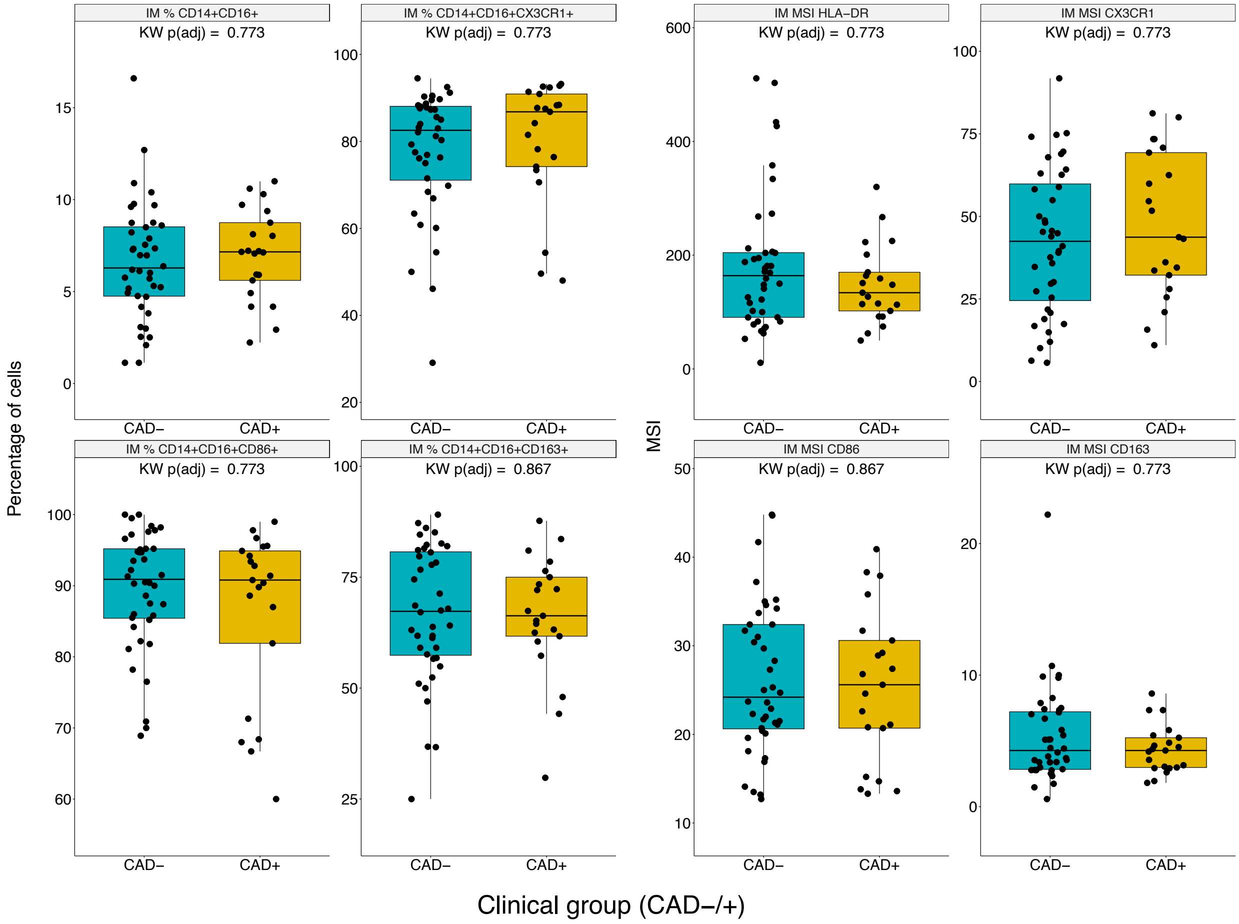

Statistical differences between CAD-/+ across intermediate monocyte (IM) populations and MFI of selected markers. Kruskal-Wallis (KW) tests were performed, and *P*-values were for False Discovery Rate (FDR) adjusted. No statistically significant differences were found.

Cobeña-Reyes J, Wanjalla CN, Feria MG, Simmons J , Temu T , Nochowicz C , Arafat SA, Kityo C , Erem G , Longenecker CT, Andorf S, Huaman MA. Characterization of Distinct Monocyte Subtypes and Immune Features Associated with HIV, Tuberculosis, and Coronary Artery Disease in a Ugandan Cohort Using Mass Cytometry. *Pathogens and Immunity*. 2025;11(1):14-38. doi: 10.20411/pai.v11i1.945

PATHOGENS AND IMMUNITY
IN ASSOCIATION WITH CASE WESTERN RESERVE UNIVERSITY AND THE RICHARD J. FASENMYER FOUNDATION

Supplementary Figure 12. Differences in non-classical monocytes manually gated populations and MFI across CAD-/+.

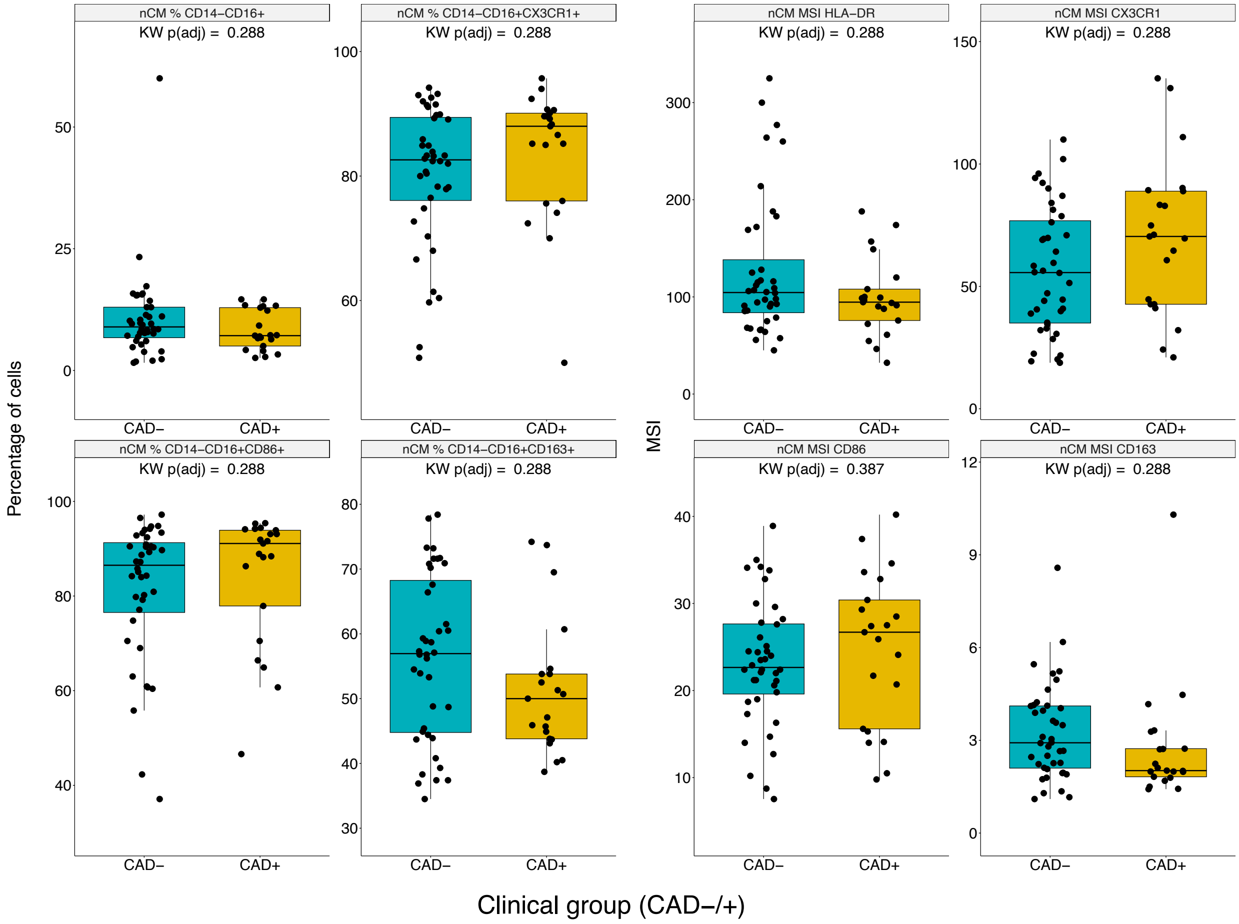

Statistical differences between CAD-/+ across non classical monocyte (nCM) populations and MFI of selected markers. Kruskal-Wallis (KW) tests were performed, and *P*-values were False Discovery Rate (FDR) adjusted. No statistically significant differences were found (all FDR-adjusted $P \geq 0.05$).

Cobeña-Reyes J, Wanjalla CN, Feria MG, Simmons J , Temu T , Nochowicz C , Arafat SA, Kityo C , Erem G , Longenecker CT, Andorf S, Huaman MA. Characterization of Distinct Monocyte Subtypes and Immune Features Associated with HIV, Tuberculosis, and Coronary Artery Disease in a Ugandan Cohort Using Mass Cytometry. *Pathogens and Immunity*. 2025;11(1):14-38. doi: 10.20411/pai.v11i1.945

PATHOGENS AND IMMUNITY
In Association with Case Western Reserve University and The Richard J. Fasenmyer Foundation

Supplementary Figure 13. Differences in total monocytes manually gated populations and MFI across HIV/TB.

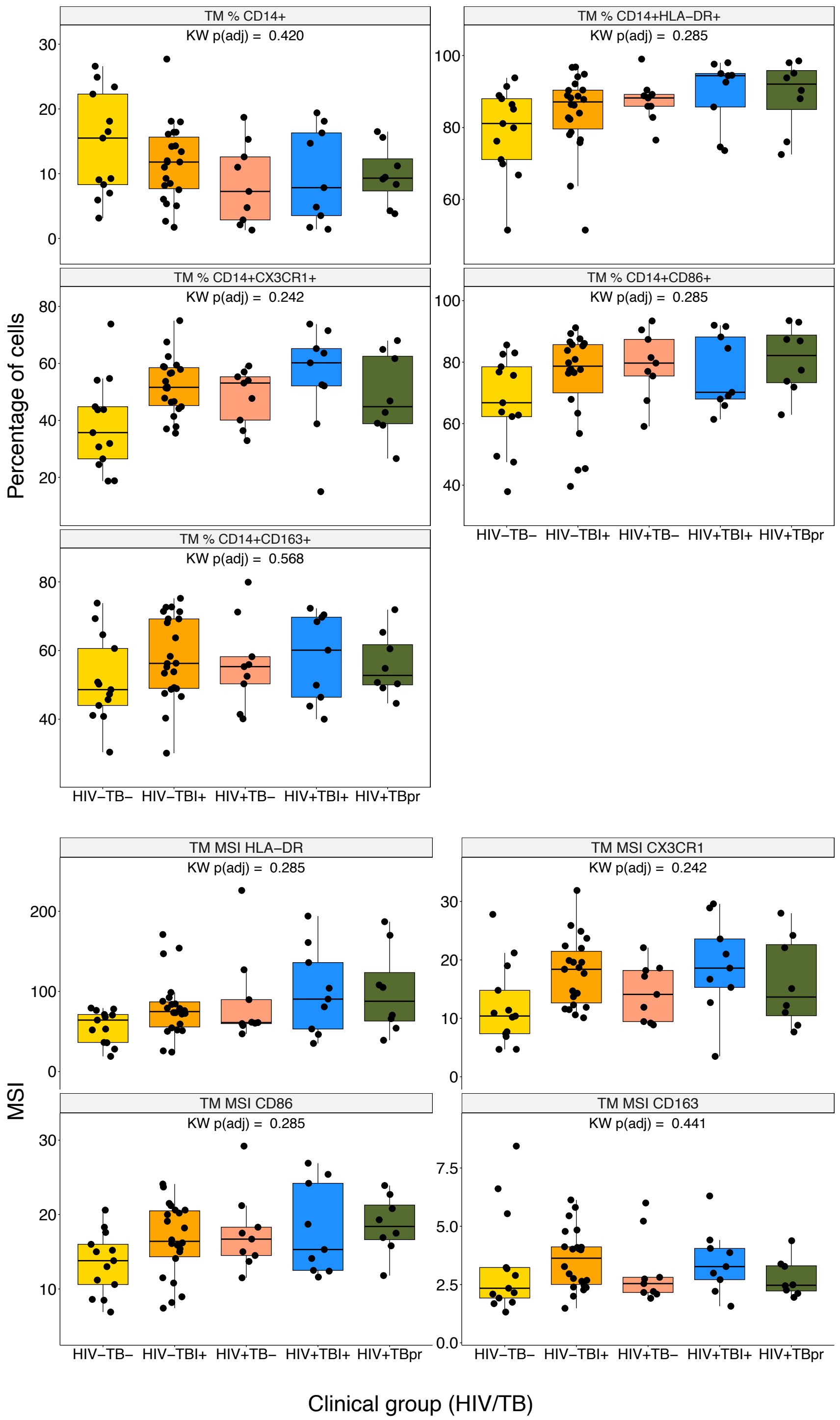

Statistical differences among HIV/TB study groups across total monocyte (TM) populations and MSI of selected markers. Kruskal-Wallis (KW) tests were performed, and p-values were False Discovery Rate (FDR) adjusted. No statistically significant differences were found.

Cobeña-Reyes J, Wanjalla CN, Feria MG, Simmons J , Temu T , Nochowicz C , Arafat SA, Kityo C , Erem G , Longenecker CT, Andorf S, Huaman MA. Characterization of Distinct Monocyte Subtypes and Immune Features Associated with HIV, Tuberculosis, and Coronary Artery Disease in a Ugandan Cohort Using Mass Cytometry. *Pathogens and Immunity*. 2025;11(1):14-38. doi: 10.20411/pai.v11i1.945

PATHOGENS AND IMMUNITY
IN ASSOCIATION WITH CASE WESTERN RESERVE UNIVERSITY AND THE RICHARD J. FASENMYER FOUNDATION

Supplementary Figure 14. Differences in classical monocytes manually gated populations and MFI across HIV/TB.

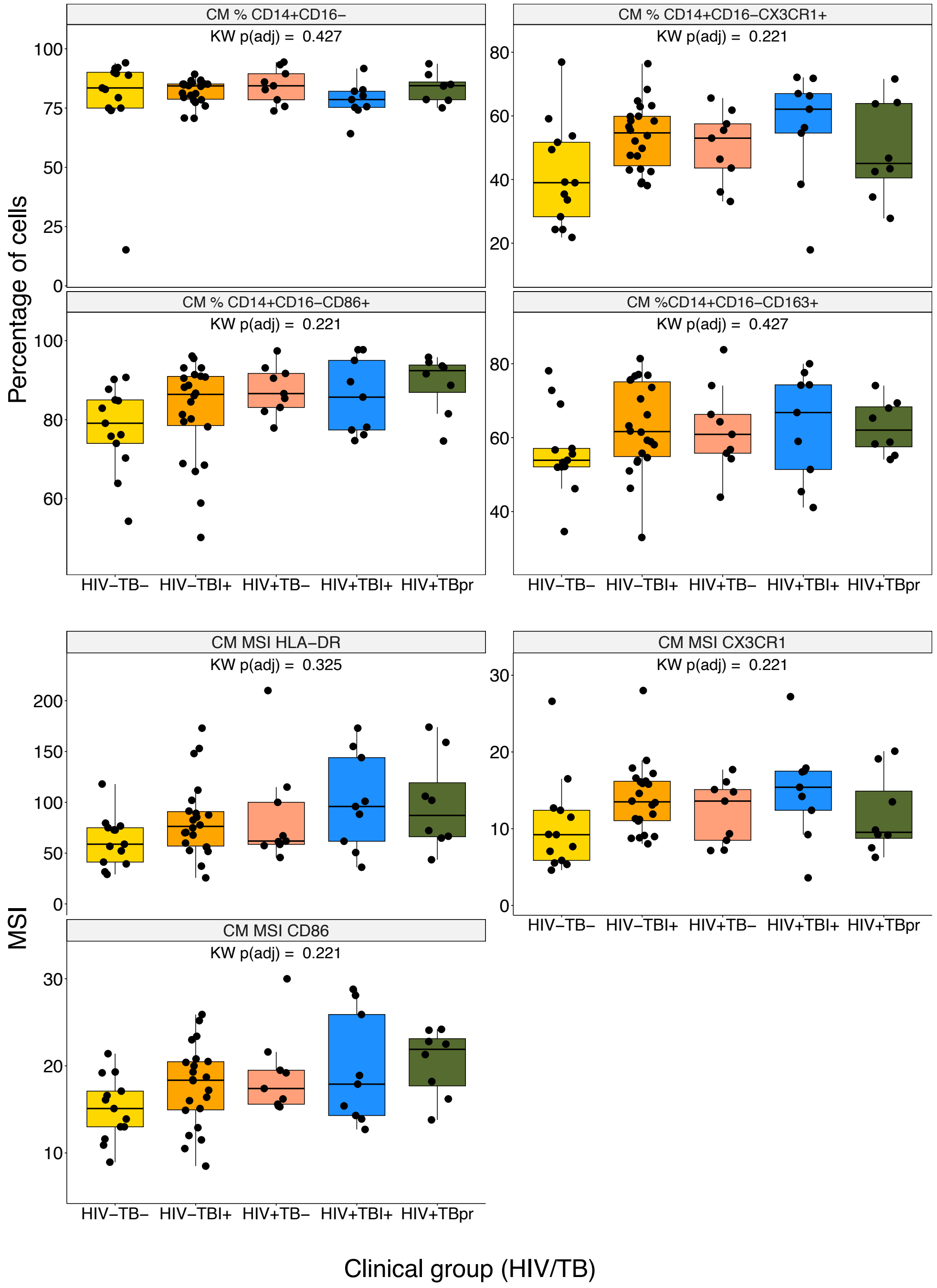

Statistical differences among HIV/TB across classical monocyte (CM) populations and MSI of selected markers. Kruskal-Wallis (KW) tests were performed, and *P*-values were False Discovery Rate (FDR) adjusted. No statistically significant differences were found.

Cobeña-Reyes J, Wanjalla CN, Feria MG, Simmons J , Temu T , Nochowicz C , Arafat SA, Kityo C , Erem G , Longenecker CT, Andorf S, Huaman MA. Characterization of Distinct Monocyte Subtypes and Immune Features Associated with HIV, Tuberculosis, and Coronary Artery Disease in a Ugandan Cohort Using Mass Cytometry. *Pathogens and Immunity*. 2025;11(1):14-38. doi: 10.20411/pai.v11i1.945

Supplementary Figure 15. Differences in intermediate monocytes manually gated populations and MFI across HIV/TB.

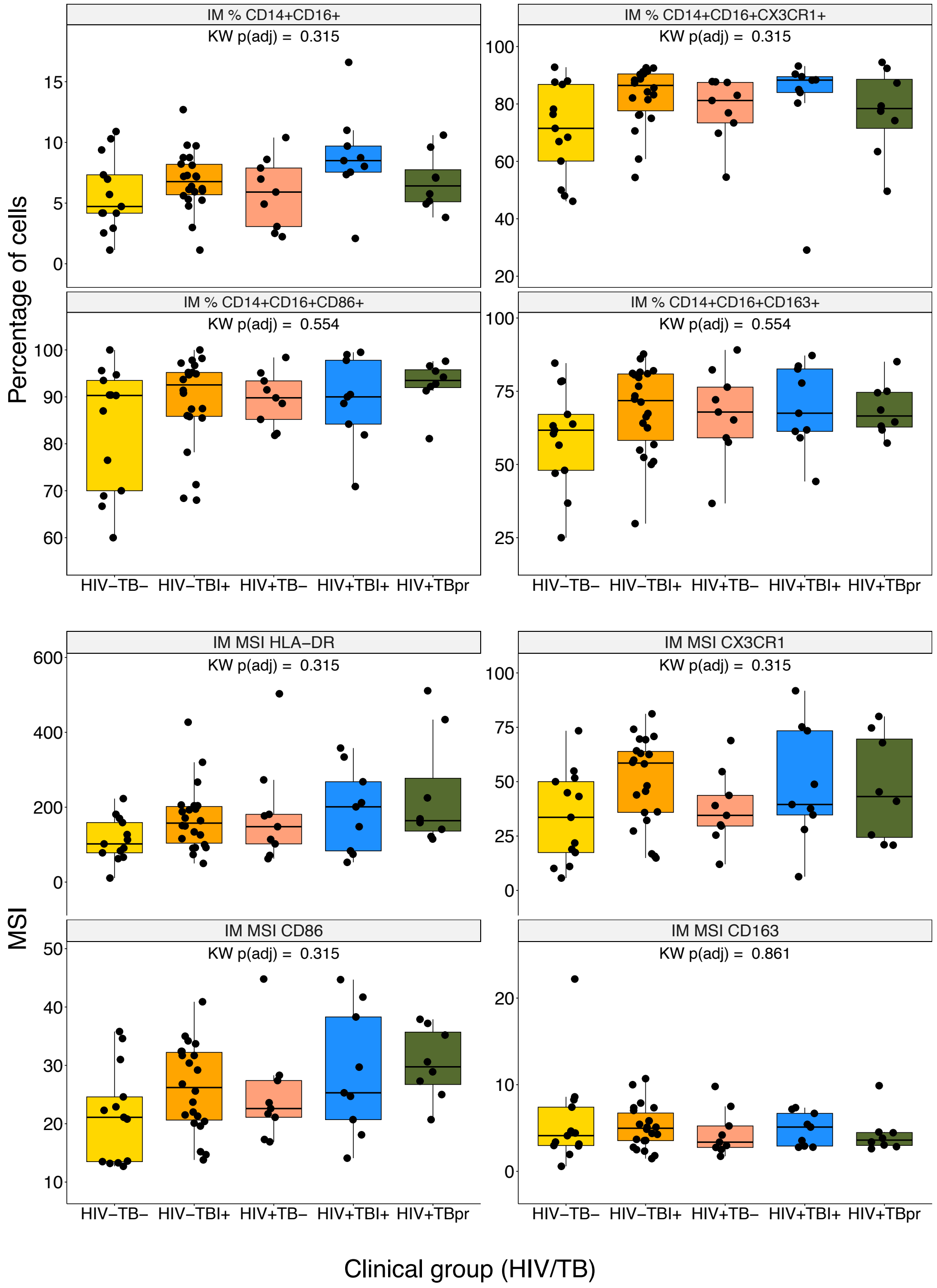

Statistical differences among HIV/TB across intermediate monocyte (IM) populations and MSI of selected markers. Kruskal-Wallis (KW) tests were performed, and *P*-values were False Discovery Rate (FDR) adjusted. No statistically significant differences were found.

Cobeña-Reyes J, Wanjalla CN, Feria MG, Simmons J , Temu T , Nochowicz C , Arafat SA, Kityo C , Erem G , Longenecker CT, Andorf S, Huaman MA. Characterization of Distinct Monocyte Subtypes and Immune Features Associated with HIV, Tuberculosis, and Coronary Artery Disease in a Ugandan Cohort Using Mass Cytometry. *Pathogens and Immunity*. 2025;11(1):14-38. doi: 10.20411/pai.v11i1.945

PATHOGENS AND IMMUNITY
IN ASSOCIATION WITH CASE WESTERN RESERVE UNIVERSITY AND THE RICHARD J. FASENMYER FOUNDATION

Supplementary Figure 16. Differences in non-classical monocytes manually gated populations and MFI across HIV/TB.

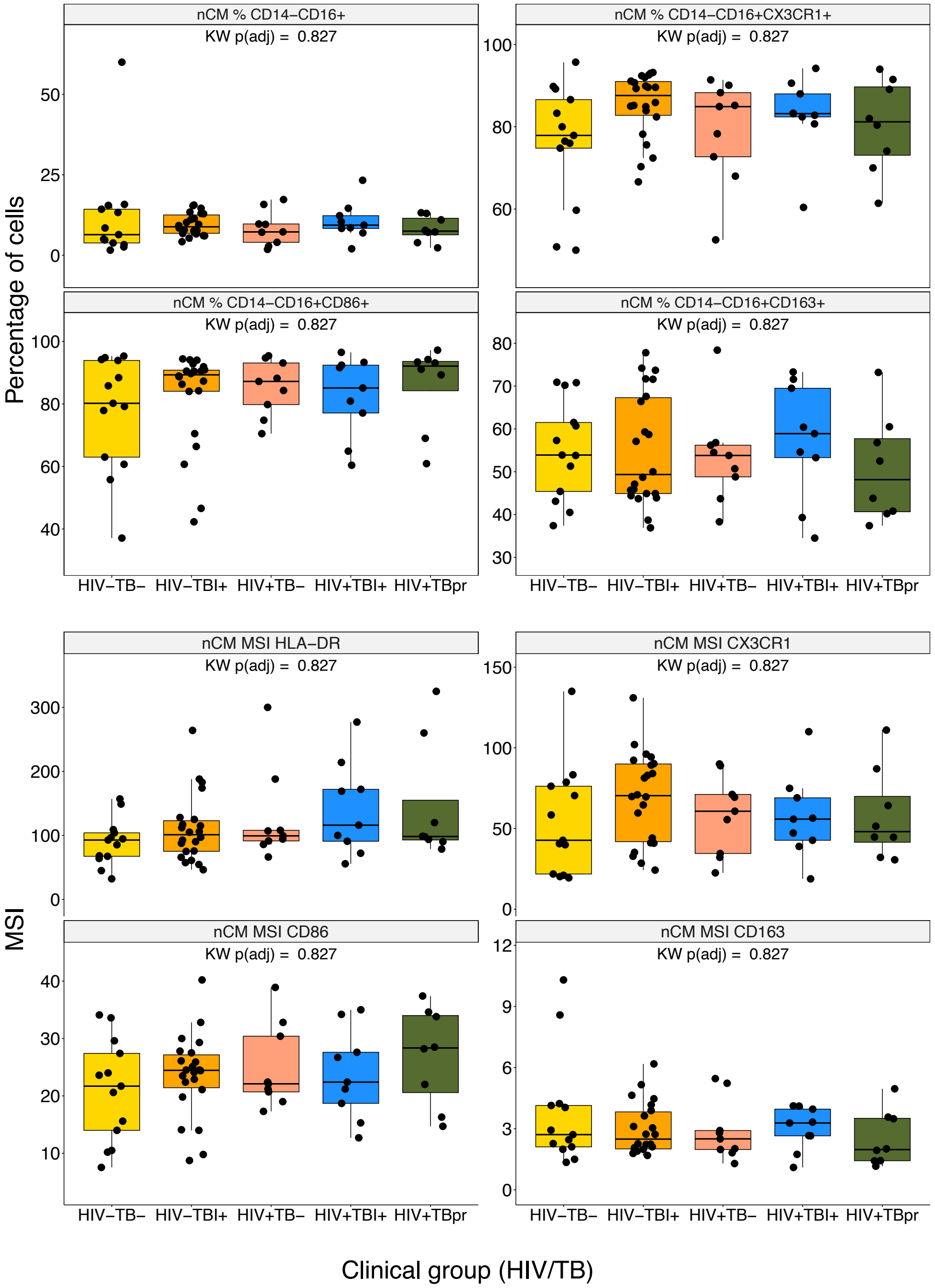

Statistical differences among HIV/TB across non classical monocyte (nCM) populations and MSI of selected markers. Kruskal-Wallis (KW) tests were performed, and p-values were False Discovery Rate (FDR) adjusted. No statistically significant differences were found.

Cobeña-Reyes J, Wanjalla CN, Feria MG, Simmons J , Temu T , Nochowicz C , Arafat SA, Kityo C , Erem G , Longenecker CT, Andorf S, Huaman MA. Characterization of Distinct Monocyte Subtypes and Immune Features Associated with HIV, Tuberculosis, and Coronary Artery Disease in a Ugandan Cohort Using Mass Cytometry. *Pathogens and Immunity*. 2025;11(1):14-38. doi: 10.20411/pai.v11i1.945

PATHOGENS AND IMMUNITY
IN ASSOCIATION WITH CASE WESTERN RESERVE UNIVERSITY AND THE RICHARD J. FASENMYER FOUNDATION

Supplementary Figure 17. Scaled median marker expressions of the monocyte populations found using FlowSOM.

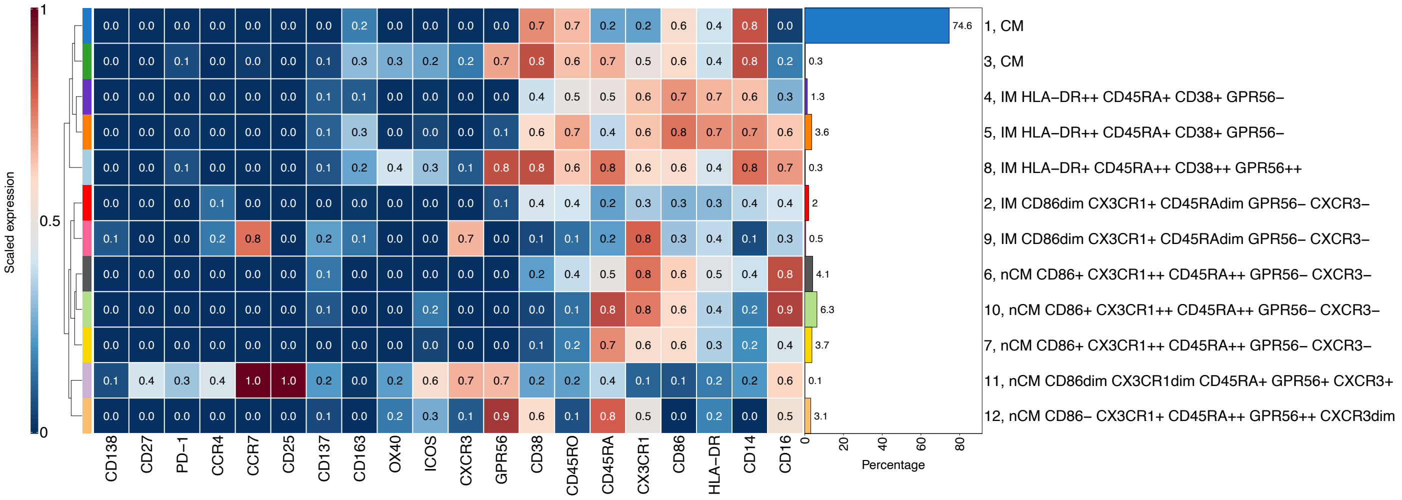

Based on CD14 and CD16 expression, the 12 populations were classified as 2 classical (CM), 5 intermediate (IM), and 5 non-classical (nCM) monocytes. The bar plot on the right indicates the percentage of the cluster with respect to all cells, i.e. manually gated monocytes, in all the samples. The cluster numbers and marker definitions per cluster are shown on the right. Clusters 1 and 3 (CM), clusters 4 and 5 (IM), clusters 2 and 9 (IM), and clusters 6, 7 and 10 (nCM) were merged due similar characteristics.

Cobeña-Reyes J, Wanjalla CN, Feria MG, Simmons J , Temu T , Nochowicz C , Arafat SA, Kityo C , Erem G , Longenecker CT, Andorf S, Huaman MA. Characterization of Distinct Monocyte Subtypes and Immune Features Associated with HIV, Tuberculosis, and Coronary Artery Disease in a Ugandan Cohort Using Mass Cytometry. *Pathogens and Immunity*. 2025;11(1):14-38. doi: 10.20411/pai.v11i1.945

PATHOGENS AND IMMUNITY
IN ASSOCIATION WITH CASE WESTERN RESERVE UNIVERSITY AND THE RICHARD J. FASENMYER FOUNDATION

Supplementary Figure 18. **Differences in monocyte subset obtained with FlowSOM across CAD-/+ groups.**

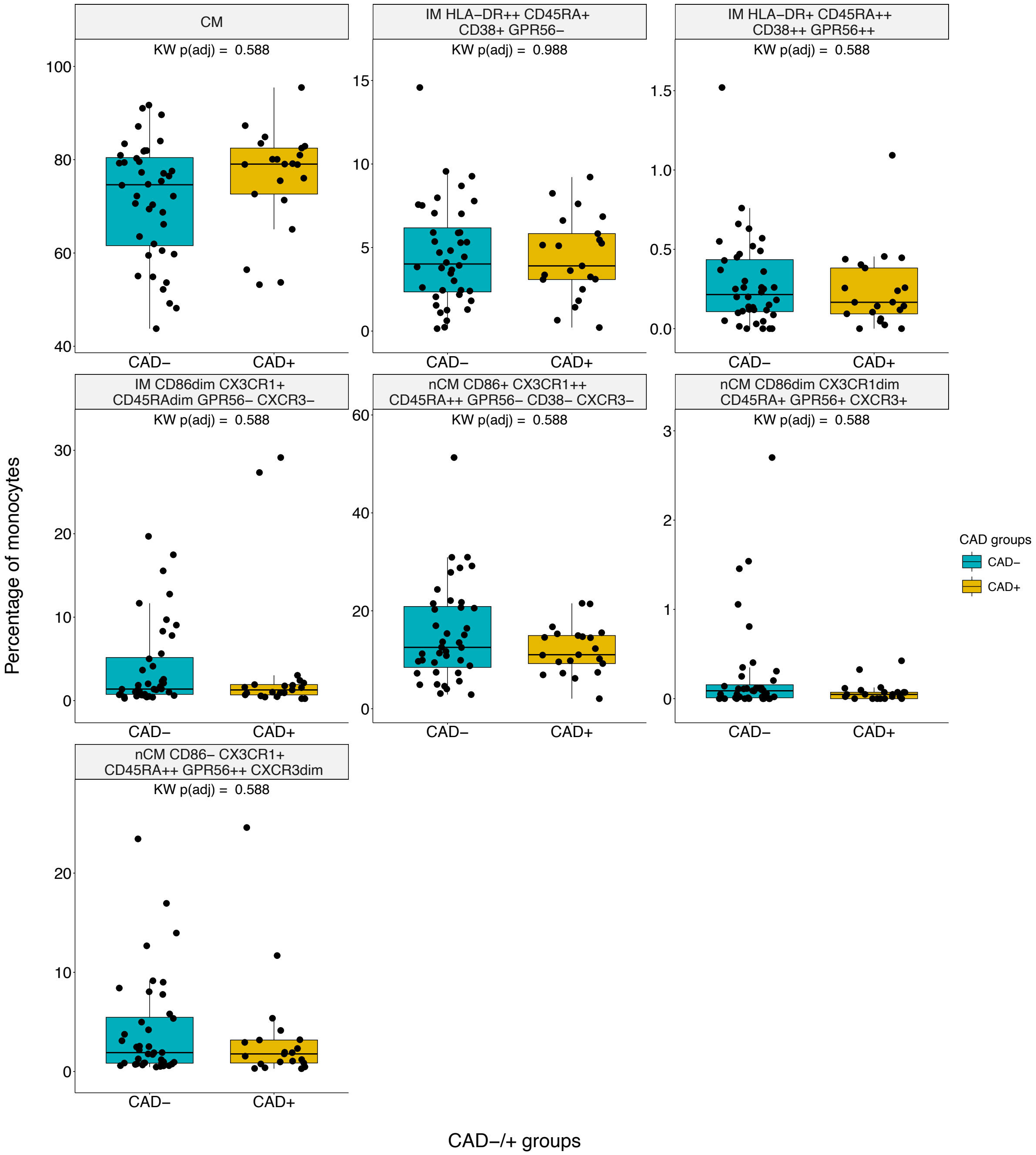

Statistical differences of percentages of cells for each monocyte population obtained from unsupervised clustering by FlowSOM across the three CAD-/+ study groups. Kruskal-Wallis (KW) tests were used as omnibus test and p-values were False Discovery Rate (FDR) adjusted. No statistical differences were found all FDR-adjusted $P \geq 0.05$.

Cobeña-Reyes J, Wanjalla CN, Feria MG, Simmons J , Temu T , Nochowicz C , Arafat SA, Kityo C , Erem G , Longenecker CT, Andorf S, Huaman MA. Characterization of Distinct Monocyte Subtypes and Immune Features Associated with HIV, Tuberculosis, and Coronary Artery Disease in a Ugandan Cohort Using Mass Cytometry. *Pathogens and Immunity*. 2025;11(1):14-38. doi: 10.20411/pai.v11i1.945

PATHOGENS AND IMMUNITY
IN ASSOCIATION WITH CASE WESTERN RESERVE UNIVERSITY AND THE RICHARD J. FASENMYER FOUNDATION

Supplementary Figure 19. Uniform Manifold Approximation and Projections (UMAPs) stratified by HIV/TB clinical groups.

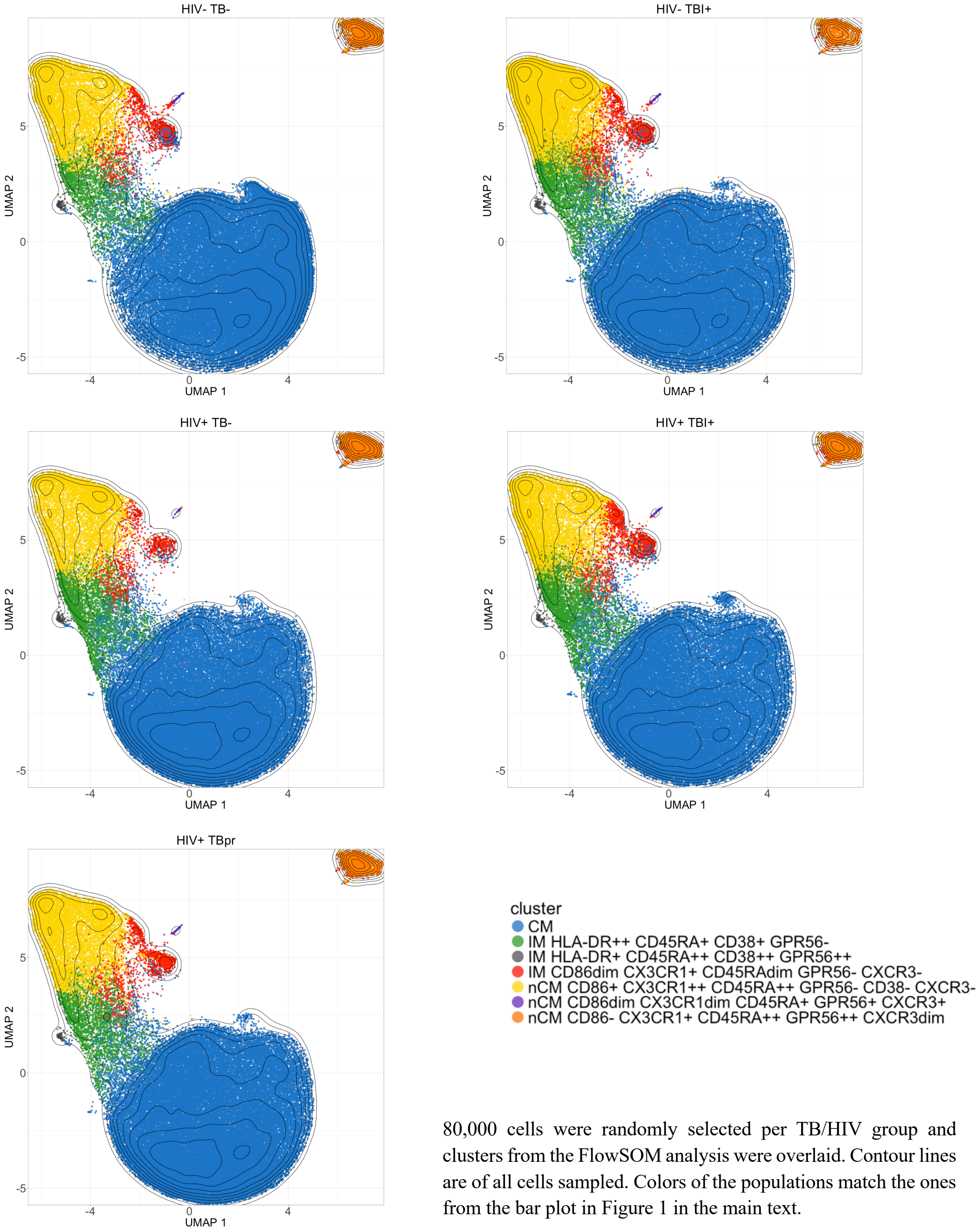

80,000 cells were randomly selected per TB/HIV group and clusters from the FlowSOM analysis were overlaid. Contour lines are of all cells sampled. Colors of the populations match the ones from the bar plot in Figure 1 in the main text.

Cobeña-Reyes J, Wanjalla CN, Feria MG, Simmons J, Temu T, Nochowicz C, Arafat SA, Kityo C, Erem G, Longenecker CT, Andorf S, Huaman MA. Characterization of Distinct Monocyte Subtypes and Immune Features Associated with HIV, Tuberculosis, and Coronary Artery Disease in a Ugandan Cohort Using Mass Cytometry. *Pathogens and Immunity*. 2025;11(1):14-38. doi: 10.20411/pai.v11i1.945

Supplementary Figure 20. Misclassification error as a function of the log of lambda value (bottom x-axis), and the number of non-zero coefficients (upper x-axis).

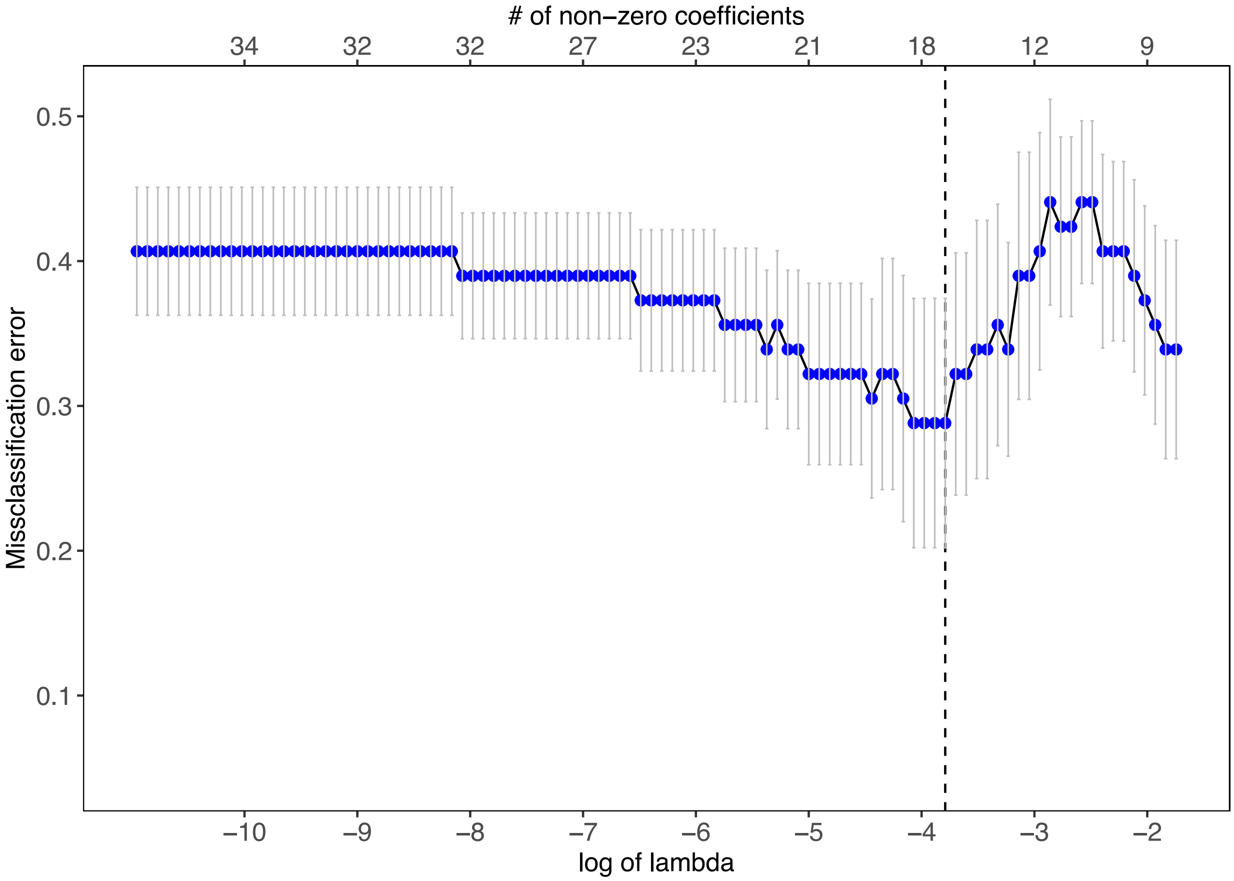

Dashed line represents the log of lambda that yields the smallest Misclassification error when alpha=0.6 (elastic net regularization). The error bars represent the standard error of the 10-fold cross-validated Misclassification error at each value of lambda. A sequence of one hundred values of lambda was tested, generated using the default settings of the glmnet package. A total of eleven values of alpha (0 – 1 in 0.1 increments) were tested. The non-zero coefficients are plotted in Figure 4 in the main text.

Cobeña-Reyes J, Wanjalla CN, Feria MG, Simmons J , Temu T , Nochowicz C , Arafat SA, Kityo C , Erem G , Longenecker CT, Andorf S, Huaman MA. Characterization of Distinct Monocyte Subtypes and Immune Features Associated with HIV, Tuberculosis, and Coronary Artery Disease in a Ugandan Cohort Using Mass Cytometry. *Pathogens and Immunity*. 2025;11(1):14-38. doi: 10.20411/pai.v11i1.945
